# Microbial Turnover and Dispersal Events Occur in Synchrony with Plant Phenology in the Perennial Evergreen Tree Crop *Citrus sinensis*

**DOI:** 10.1128/mbio.00343-22

**Published:** 2022-06-01

**Authors:** Nichole A. Ginnan, N. Itzel De Anda, Flavia Campos Freitas Vieira, Philippe E. Rolshausen, M. Caroline Roper

**Affiliations:** a Department of Microbiology and Plant Pathology, University of California, Riverside, California, USA; b Department of Botany and Plant Sciences, University of California, Riverside, California, USA; University of Nebraska-Lincoln

**Keywords:** *Citrus sinensis*, dispersal, evergreen, flowering, fruiting, microbiome, perennial, plant phenology, DNA sequencing, environmental microbiology, microbial communities, microbial ecology, plant-microbe interactions

## Abstract

Emerging research indicates that plant-associated microbes can alter plant developmental timing. However, it is unclear if host phenology affects microbial community assembly. Microbiome studies in annual or deciduous perennial plants face challenges in separating effects of tissue age from phenological driven effects on the microbiome. In contrast, evergreen perennial trees, like *Citrus sinensis*, retain leaves for years, allowing for uniform sampling of similarly aged leaves from the same developmental cohort. This aids in separating phenological effects on the microbiome from impacts due to annual leaf maturation/senescence. Here, we used this system to test the hypothesis that host phenology acts as a driver of microbiome composition. *Citrus sinensis* leaves and roots were sampled during seven phenological stages. Using amplicon-based sequencing, followed by diversity, phylogenetic, differential abundance, and network analyses, we examined changes in bacterial and fungal communities. Host phenological stage is the main determinant of microbiome composition, particularly within the foliar bacteriome. Microbial enrichment/depletion patterns suggest that microbial turnover and dispersal were driving these shifts. Moreover, a subset of community shifts were phylogenetically conserved across bacterial clades, suggesting that inherited traits contribute to microbe-microbe and/or plant-microbe interactions during specific phenophases. Plant phenology influences microbial community composition. These findings enhance understanding of microbiome assembly and identify microbes that potentially influence plant development and reproduction.

## INTRODUCTION

Plant phenology, the periodic timing of plant life cycle events, is innately linked to exogenous climatic variables that affect plant development, such as temperature, photoperiod, and nutrient and water availability, as well as other abiotic and biotic factors (1, 2). Additionally, endogenous genome-encoded factors such as dynamic internal photosynthate source-sink pathways, intricate phytohormone signaling networks, and other developmental regulatory processes mediate the transition between phenological stages (3, 4). The timing of specific developmental stages, such as flowering, can determine a plant’s geographic distribution range as well as determine crop yield and productivity (5, 6). Alterations in plant phenology can also have a cascading effect on the fitness of organisms that depend on those specific plants for nutrient acquisition, such as pollinator species (7–9).

Citrus is a significant economic crop and provides several health benefits because of the myriad of nutrients, antioxidants, vitamins, minerals, and dietary fiber found in fresh and juiced citrus fruits (10–12). Citrus varieties are grown across the globe, and because of this, citrus phenology is well characterized to guide management strategies of different varieties for specific climatic conditions. Phenological modeling of citrus has focused primarily on buds, flowers, and fruit and is used to predict bloom time across different growing regions (13). This has implications for protecting flowers from floral pests and pathogens by allowing growers to time spray applications in an informed manner (14). Citrus flowers are a significant source of nectar related to honey production, particularly in California’s Central Valley. As such, bloom timing models are also important for the beekeeping industry (15). In addition, bloom time and duration models can be extrapolated to predict fruit set (16), and these performance models can provide yield predictions.

Soil and rhizosphere microbiomes can drive changes in flowering time in the herbaceous perennial plant Boechera stricta, a wild relative of *Arabidopsis* (17, 18), and affect other aboveground plant traits in the annual plant system Brassica rapa (19, 20). However, questions about microbiome shifts associated with transitions between phenological stages have not been addressed in perennial trees, particularly domesticated evergreens like citrus (21). Citrus phenology models primarily take into account temperature and number of degree days above a certain threshold temperature (16) but, to the best of our knowledge, have not incorporated studies on the microbial communities associated with transitions across phenological stages. The citrus microbiome is an emerging prototype for understanding microbial contributions to plant health in a perennial arboreal crop system (22–26). Due to its well-defined phenology, citrus is an ideal system to investigate the interplay between host phenology and microbial community composition.

Several seminal studies in annual and short-lived perennial plants have characterized changes in rhizosphere and root microbiome composition across plant developmental cycles, suggesting that host phenology drives these alterations (27–32). However, Dombrowski et al. suggests that initially microbiota are sequentially acquired resulting in community changes as the host ages but eventually the microbiome matures and stabilizes, functioning independently from host development (33). Another recent study supports the idea that time is a stronger predictor of microbiome composition than plant developmental stage (34). This prompts discussion on whether these community shifts are a consequence of tissue age and a microbiome maturation process or if these changes are driven by plant phenology. In addition to producing and maturing leaves and roots throughout the year, long-lived evergreen perennial plant systems retain mature leaves for 1 to 3 years (35, 36), which allows for selection of leaf tissues of similar age and developmental cohort across phenophases. Because of these features, we utilized this system to help decouple tissue age from host phenological effects and tested the hypothesis that host phenology acts as a driver of community compositional shifts within the aboveground (foliar) and belowground (root) microbiomes of citrus. Indeed, we determined that the significant shifts in both diversity and composition of the microbial community structure were driven primarily by host phenological stages and not exogenous environmental factors such as rainfall, hours of irrigation, or temperature. Foliar communities were more affected by host phenology than root microbiomes, which were comparably more stable. Interestingly, major alterations in foliar microbial community composition correlated with the shifts in source-to-sink pathways of carbohydrate transit, namely, during the transition from floral bud development to full flowering to fruit set. More specifically, subsets of these taxa displayed temporal turnover patterns indicating that specific taxa were enriched as trees shifted to reproductive growth associated with fruit production. We also observed taxa typically associated with pollinator species that were substantially enriched only during flowering, suggesting that these microbes were introduced into the foliar microbiome as microbial immigrants via an insect-mediated dispersal mechanism.

In agricultural plant systems, comprehensive microbiome studies allow researchers to place an emphasis on how the microbiome as a whole functions to promote overall plant health by a variety of mechanisms, such as enhancing nutrient uptake or resisting pathogen ingress to promote a sustainable agroecosystem. Uncovering links between plant phenology and shifts in microbiome structure is the first step toward a mechanistic understanding of microbiome resilience over cyclical development in a perennial plant host. In addition, this can further serve as the foundation to understanding how the microbiome responds to changes in host development and, in turn, if microbiome community structure can influence host phenological transitions.

## RESULTS

### Significant shifts in alpha diversity occur across phenological stages.

**FIG 1:**
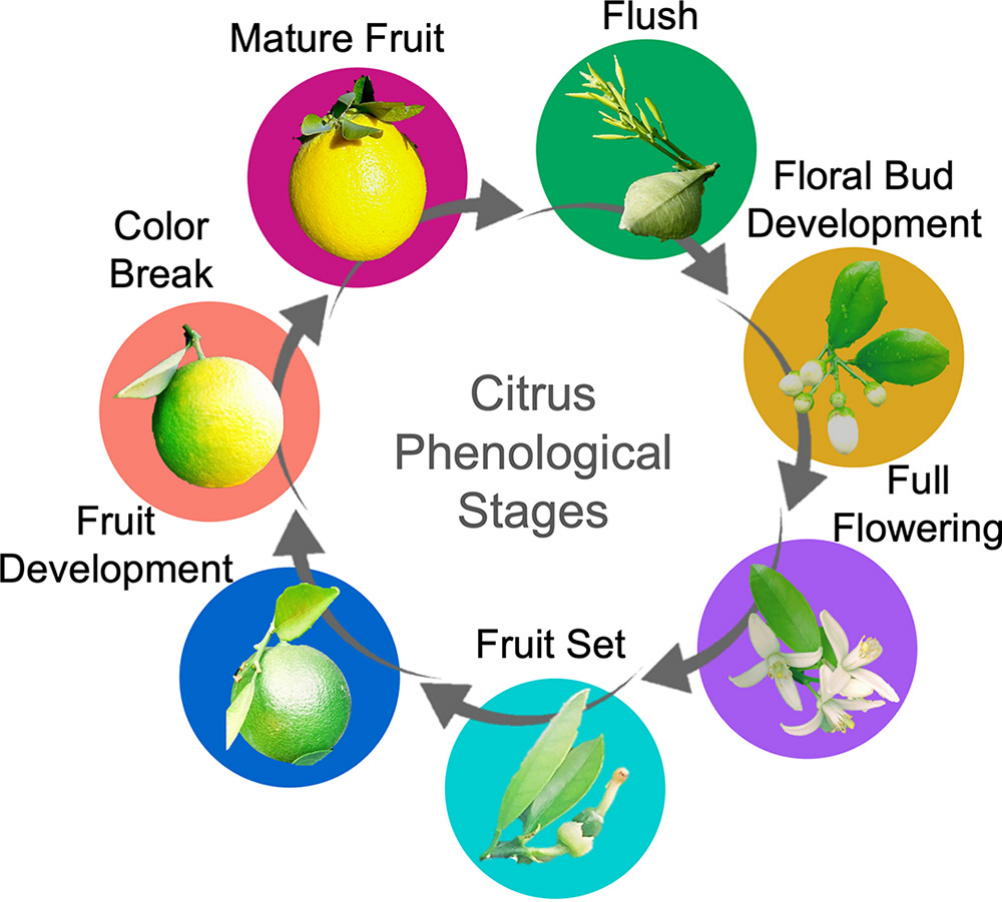
Citrus phenological stages. Cyclic seasonal development of *Citrus sinensis*.

**FIG 2:**
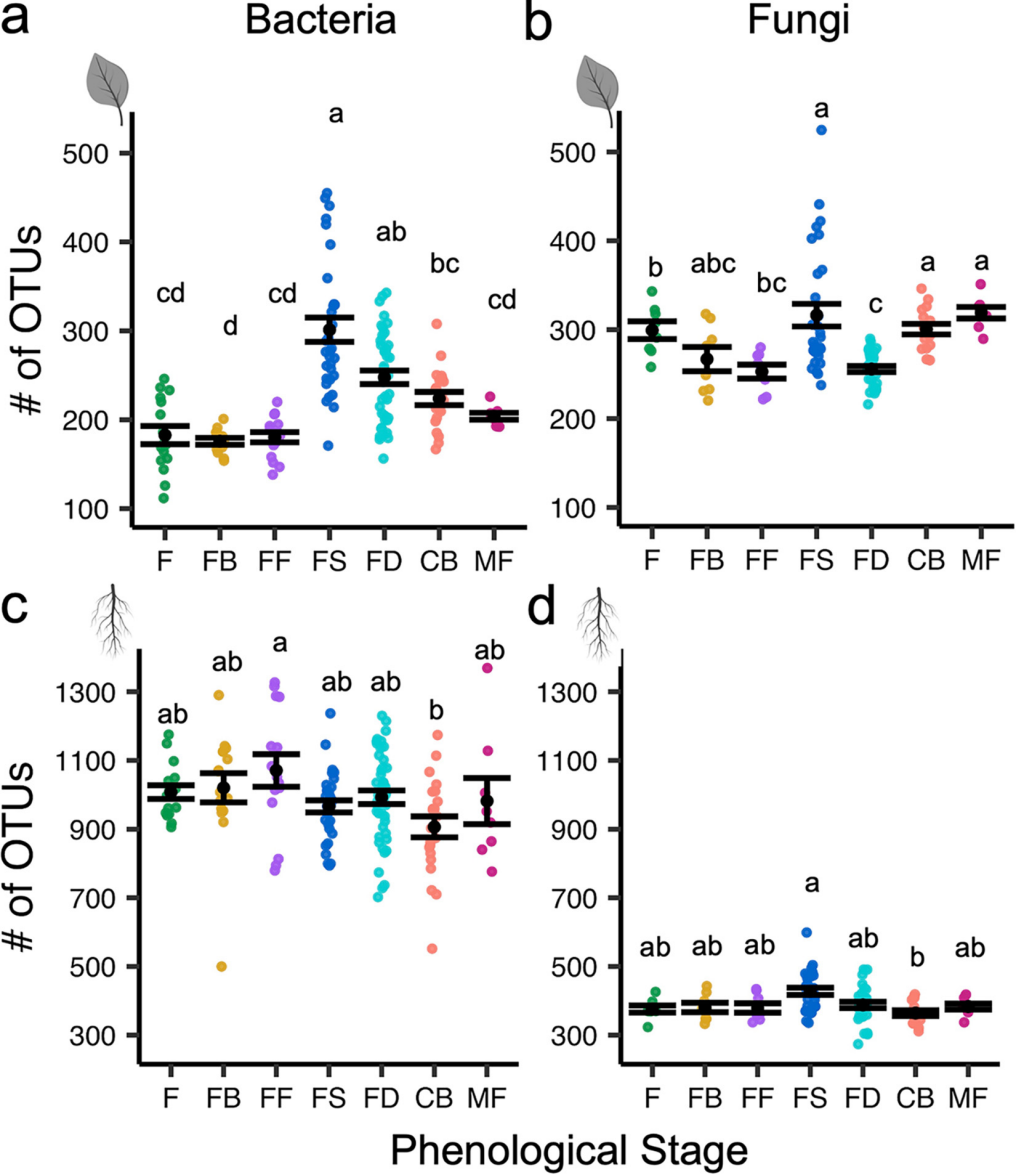
Species richness varies across phenophases. Alpha diversity plot of richness (no. of OTUs) for individual sampling events for bacterial leaf (a), fungal leaf (b), bacterial root (c), and fungal root (d) communities. Black points, “•,” represent the mean, and error bars represent standard error. Letters indicate a significant difference of *P ≤ *0.05, determined using a Kruskal-Wallis test, with a pairwise Dunn’s test and correcting for multiple comparisons with Holm’s method. Phenological stages on the *x* axis include flush (F), floral bud break (FB), full flowering (FF), fruit set (FS), fruit development (FD), color break (CB), and mature fruit (MF).

We focused our study on seven citrus phenophases that included spring vegetative shoot flush, referred to as “flush” (F), early floral bud break and development (FB), full flowering (FF), fruit set (FS), exponential fruit growth and development, referred to as “fruit development” (FD), color break (CB; initiation of fruit maturation), and mature fruit (MF). Citrus phenological stages can overlap on individual trees, and some stages span multiple months; thus, some stages include multiple months of sampling (Fig. 1 and see Fig. S1 in the supplemental material). Initially, we accessed alpha diversity or the diversity within a sample, in this case the number of operational taxonomic units (OTUs). Overall, bacterial and fungal leaf microbiomes had the most significant shifts in alpha diversity across phenological stages compared to those of the root microbiomes. Specifically, alpha diversity in both the leaf bacteriome and mycobiome remained consistent as trees transitioned from leaf flush to flowering (floral bud development and full flowering). Following full flowering, there was a significant increase in alpha diversity in the leaf bacteriome and mycobiome at fruit set (Fig. 2a and b). Species richness within the leaf bacteriome significantly decreased when trees transitioned from fruit growth and development to color break and mature fruit stages.

10.1128/mbio.00343-22.5 FIG S1 Environmental factors fluctuate during citrus development. The primary phenophase displayed during each month from July 2017 to April 2019 is indicated by colors on the *x* axis. (a) Gray bars represent total hours of irrigation each month. (b) Navy bars represent mean total rainfall each month. (c) Points indicate average high (red), average low (blue), and total monthly average (black) temperatures. Download FIG S1, PDF file, 1.7 MB. Copyright © 2022 Ginnan et al. 2022 Ginnan et al. https://creativecommons.org/licenses/by/4.0/ This content is distributed under the terms of the Creative Commons Attribution 4.0 International license.

Despite being relatively stable across the study, root bacteriome alpha diversity peaked during full flowering. Similar to the overall leaf microbiome, the root mycobiome had the highest alpha diversity during fruit set (Fig. 2c and d). Our study did not discriminate between rhizoplane and endophytic root microbiota, nor was it possible to select feeder roots of a specific age cohort. Future work that separates these compartments in similarly aged roots may reveal more finely resolved shifts in species richness associated with these root environments.

### Host phenological stage is a major determinant of community composition.

**FIG 3:**
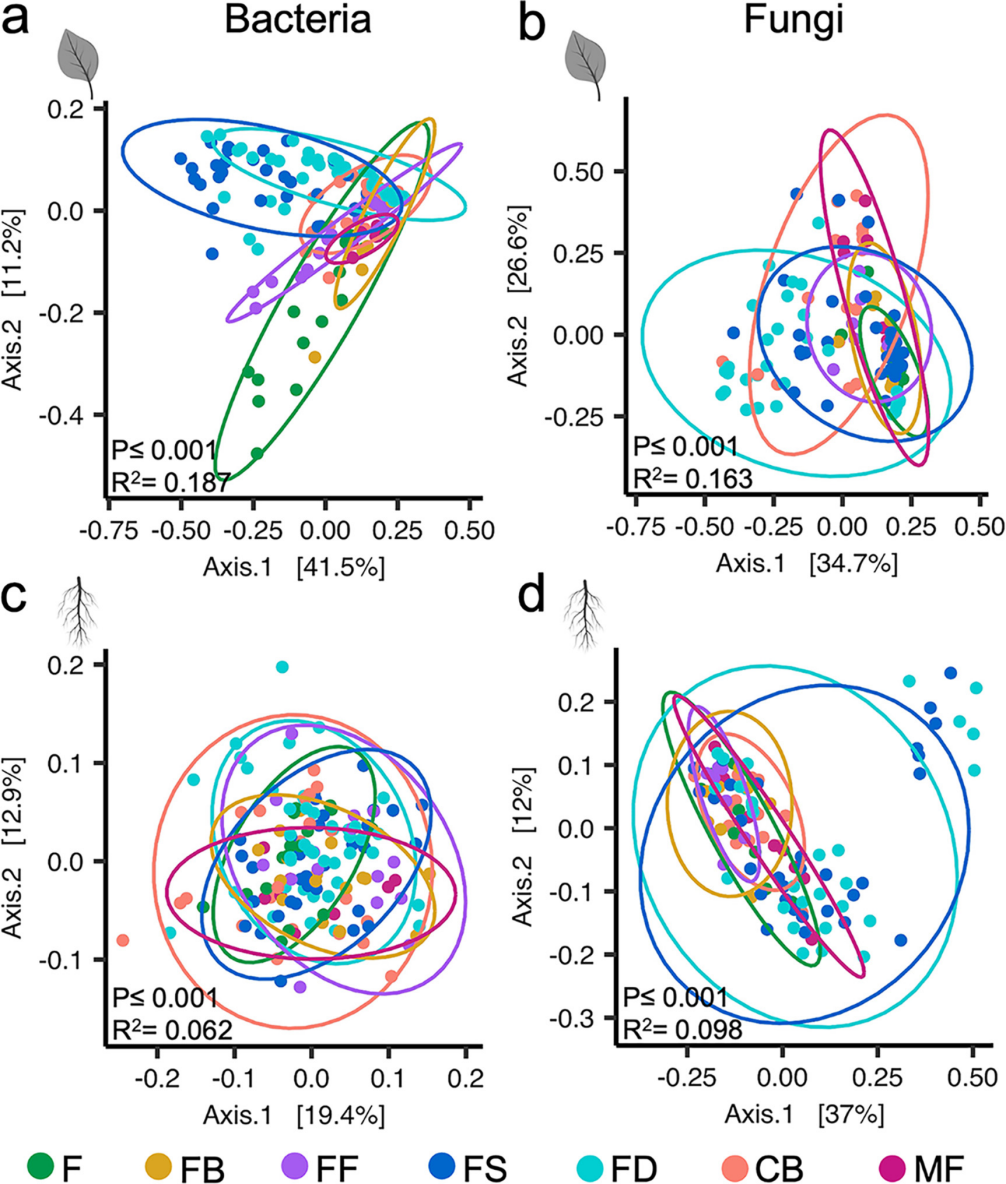
Host phenology affects community diversity and composition. Beta diversity PCoA plots of bacterial leaf (a), fungal leaf (b), bacterial root (c), and fungal root (d) communities. Points are colored by phenological stage and represent a complete community from a single leaf or root sample. Ellipses represent 95% confidence intervals. The *P* values and *r^2^* values were obtained using a PERMANOVA (Adonis). Phenological stages include flush (F), floral bud break (FB), full flowering (FF), fruit set (FS), fruit development (FD), color break (CB), and mature fruit (MF).

Although climatic variables can be difficult to uncouple from plant development variables, the greatest amount of the variation in the data was attributed to the host phenological stage for all four communities (permutational multivariate analysis of variance [PERMANOVA], *P* ≤ 0.001, *r^2^* = 0.062 to 0.187) (Table 1, Fig. 3). Time (i.e., sample year) had less impact than phenology on beta diversity (variation in taxa abundance and composition between samples) across all communities (Table 1, Fig. S2). Interestingly, the community composition (beta diversity) of leaf bacteriome and mycobiome was influenced by host phenology more than that of root communities, indicating that changes in host phenology had a larger influence on diversity within foliar microbiomes than in root microbiomes. Specifically, a principal coordinate analysis of weighted UniFrac distances indicated significant clustering of individual microbial communities by phenological stage (Fig. 3). In a pairwise comparison of community compositional differences between each phenological stage, the leaf bacteriome had the greatest number of significant adjusted *P* values, with 21 of the 21 pairwise comparisons being significantly different, followed by the leaf mycobiome (19 of 21 comparisons) (Table S2). Root communities had fewer significantly different pairwise comparisons (root mycobiome = 5/21, root bacteriome = 8/21).

**TABLE 1:** Beta diversity analysis (PERMANOVA) results for all variables^*a*^

Characteristic	Leaf bacteria				Leaf fungi			
	*df*	*F*	*r^2^*	*P*	*df*	*F*	*r^2^*	*P*
Phenology	**6**	**6.706**	**0.187**	**0.001**	**6**	**3.893**	**0.163**	**0.001**
Sample yr	1	6.205	0.029	0.001	1	11.065	0.077	0.001
Fertilizer	1	5.854	0.027	0.001	1	2.579	0.018	0.003
Avg. temp	1	5.984	0.028	0.001	1	1.762	0.012	0.041
Hours of irrigation	1	2.866	0.013	0.008	1	3.787	0.026	0.001
Total rain	1	3.802	0.018	0.001	1	2.072	0.014	0.023
Phenology: sample yr	5	3.557	0.082	0.001	1	2.141	0.015	0.012
Avg. temp: hours of irrigation	1	1.972	0.009	0.038	1	1.402	0.010	0.119
Residuals	130		0.605		95		0.664	

	**Root bacteria**				**Root fungi**			
Phenology	**6**	**1.676**	**0.062**	**0.001**	**6**	**2.038**	**0.098**	**0.001**
Sample yr	1	3.749	0.023	0.001	1	4.044	0.032	0.001
Fertilizer	1	1.452	0.009	0.036	1	3.890	0.031	0.001
Avg. temp	1	0.962	0.006	0.497	1	2.151	0.017	0.008
Hours of irrigation	1	2.077	0.013	0.001	1	2.072	0.017	0.011
Total rain	1	1.326	0.008	0.061	1	2.523	0.020	0.005
Phenology: sample yr	5	1.299	0.040	0.002	1	2.193	0.018	0.008
Avg. temp: hours of irrigation	1	0.986	0.006	0.483	1	1.174	0.009	0.211
Residuals	136		0.834		95		0.758	

a Test was performed using the vegan::adonis2 function and square-root transformed weighted UniFrac distances. Bold text indicates the variable with the greatest *r*^2^ value (phenology).

10.1128/mbio.00343-22.2 TABLE S2 Phenological stage pairwise PERMANOVA results (adjusted *P* values). Significant differences in beta diversity (*P ≤ *0.05) are indicated by bolded font. Phenological stages include flush (F), floral bud break (FB), full flowering (FF), fruit set (FS), fruit development (FD), color break (CB), and mature fruit (MF). Download Table S2, DOCX file, 0.01 MB. Copyright © 2022 Ginnan et al. 2022 Ginnan et al. https://creativecommons.org/licenses/by/4.0/ This content is distributed under the terms of the Creative Commons Attribution 4.0 International license.

10.1128/mbio.00343-22.6 FIG S2 Sample year has minor effects on community diversity and composition. Beta diversity PCoA plots of bacterial leaf (a) and bacterial root (b) communities. Points are colored by sample year and represent a complete community from a single leaf or root sample. Ellipses represent 95% confidence intervals. Download FIG S2, TIF file, 1.6 MB. Copyright © 2022 Ginnan et al. 2022 Ginnan et al. https://creativecommons.org/licenses/by/4.0/ This content is distributed under the terms of the Creative Commons Attribution 4.0 International license.

Rainfall, fertilizer applications, temperature, and irrigation hours fluctuated across our sampling period (Fig. S1, Table S3). Rainfall was sparse in this sample location (the Central Valley of California), ranging from 0.00 to 2.55 inches each month (Fig. S1b, Table S3), and total rainfall was a minor determinant of community structure across all four communities, explaining only 0.8 to 2.0% of the variation (PERMANOVA, *P* ≤ 0.001 to 0.061, *r^2^* = 0.008 to 0.020) (Table 1). Similarly, fertilizer application describes a small percentage (0.9 to 3.1%) of the variation in the data for all four communities examined. We evaluated temperature based on the average temperature and interactive effects it might have with water availability (hours of irrigation) in order to capture the full range of conditions that could affect microbial community composition. Temperature had a minor impact on communities, as this factor describes only 0.6 to 2.8% of the variation in the data that include temperature as an interaction factor. In addition to phenology, interactions between phenology and sample year were a driving factor of leaf bacterial community composition, explaining 8.2% of the changes across the data (PERMANOVA, *P* ≤ 0.001, *r^2^* = 0.082).

10.1128/mbio.00343-22.3 TABLE S3 Metadata file. Download Table S3, XLSX file, 0.04 MB. Copyright © 2022 Ginnan et al. 2022 Ginnan et al. https://creativecommons.org/licenses/by/4.0/ This content is distributed under the terms of the Creative Commons Attribution 4.0 International license.

Taken together, these beta diversity analyses indicate that plant phenological stage was the major driving factor in community composition for bacterial and fungal communities associated with leaves and roots. Significant compositional shifts were also visible at the phylum level, particularly in the leaf bacterial community (Fig. S3). Other covariates tested (irrigation, mean temperature, fertilizer applications, rainfall, and sample year) were minor or insignificant contributors to citrus-associated leaf and root microbiome composition.

10.1128/mbio.00343-22.7 FIG S3 Phylum-level compositional changes across phenological stages. Stacked bar plots showing relative abundance of bacterial leaf (a), fungal leaf (b), bacterial root (c), and fungal root (d) phyla across phenological stages. Phenological stages on the *x* axis include flush (F), floral bud break (FB), full flowering (FF), fruit set (FS), fruit development (FD), color break (CB), and mature fruit (MF). Download FIG S3, PDF file, 0.9 MB. Copyright © 2022 Ginnan et al. 2022 Ginnan et al. https://creativecommons.org/licenses/by/4.0/ This content is distributed under the terms of the Creative Commons Attribution 4.0 International license.

### Stable, phylogenetically conserved microbial signatures across phenophases.

**FIG 4:**
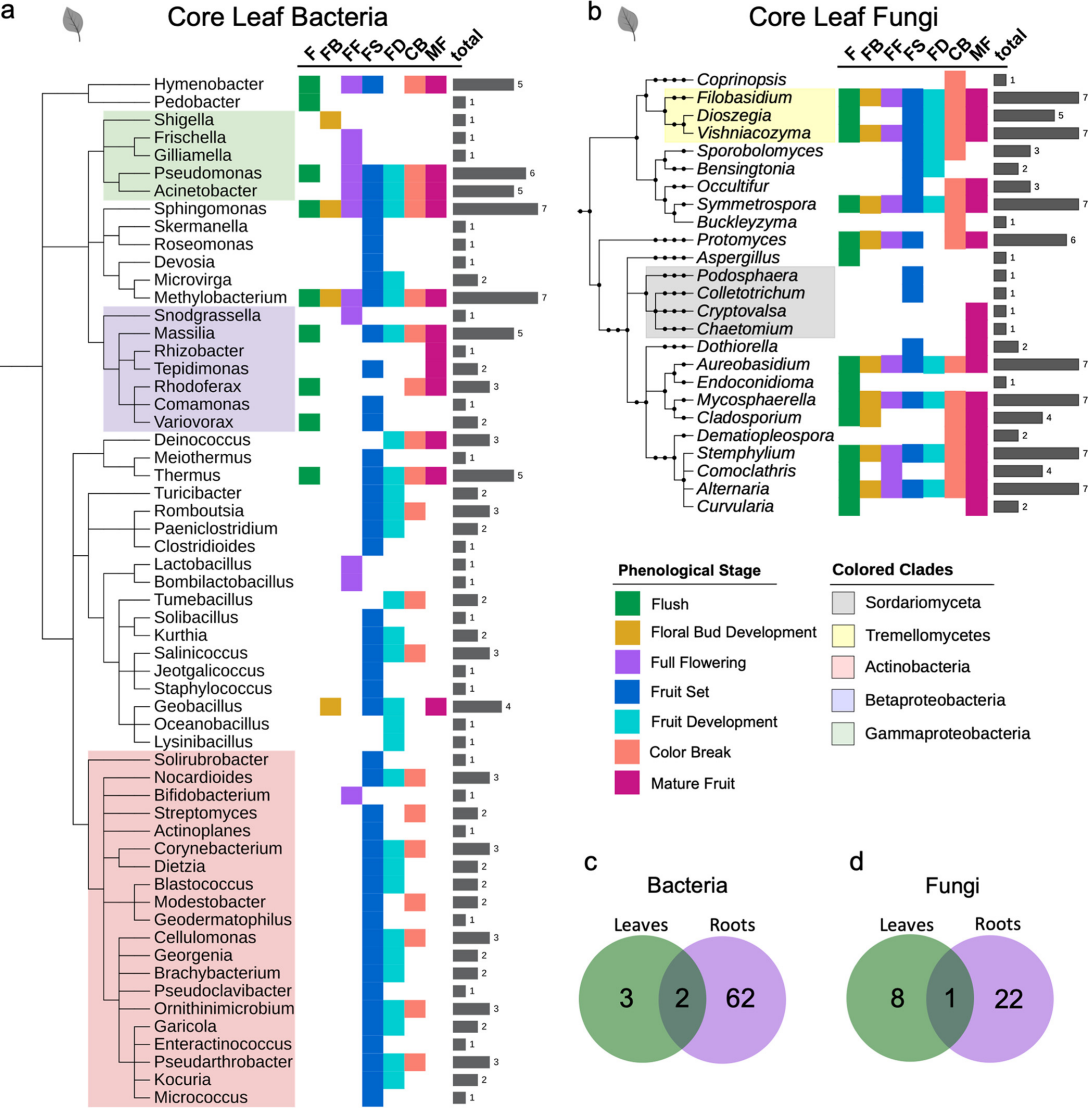
A subset of leaf bacterial and fungal core genera have phylogenetically conserved phenological association patterns. Phylogenetic trees of leaf bacterial (a) and fungal (b) genera that were core (≥0.01% relative abundance and ≥75% prevalence) to one or more stages. Colored squares indicate a genus is core to flush (green), floral bud break (gold), full flowering (purple), fruit set (blue), fruit development (light blue), color break (salmon), and/or mature fruit (magenta). Gray bars indicate the total number of phenological stages during which each genus is core. Venn diagrams show the number of highly stable core bacterial (c) and fungal (d) genera in leaf and root communities.

We identified core microbial taxa for each of our seven phenological stages. Our core bacterial and fungal leaf and fungal root microbiomes include genera that were greater than 0.01%, and core root bacteriome included genera greater than 0.1%, relative abundance in at least 75% of the samples within a phenological stage. All of our downstream analyses used genera that met our core taxa cutoffs in at least one phenophase. We assessed our core taxa and separated them into three categories: (i) high stability, defined as core member of six or more phenophases, (ii) medium stability, core member of three, four, or five phenophases, or (iii) low stability, core member of two or fewer phenophases. We determined that of the identified core there were 3 (5.2%) leaf bacterial, 8 (30.7%) leaf fungal, 62 (70.4%) root bacterial, and 22 (61.1%) root fungal core genera that had high stability across phenophases (Fig. 4, Fig. S4). This suggests that both bacterial and fungal root communities have a substantially greater number of consistent or stable microbial features across the developmental cycle. However, our experimental design did not differentiate between endophytes versus epiphytes and, thus, may have missed some fine resolution microbial community shifts occurring between the endosphere and episphere. There were two bacterial (*Pseudomonas* and *Sphingomonas*) and one fungal (*Aureobasidium*) genera that were highly stable in both roots and leaves (Fig. 4c and d).

10.1128/mbio.00343-22.8 FIG S4 Phenological core root bacterial and fungal genera. Phylogenetic trees of root bacterial (a) and fungal (b) genera that are core (≥0.01% relative abundance and ≥75% prevalence) to one or more stages. Colored squares indicate a genus is core to flush (green), floral bud break (gold), full flowering (purple), fruit set (blue), fruit development (light blue), color break (salmon), and/or mature fruit (magenta). Gray bars indicate the total number of phenological stages where that genus is core. Download FIG S4, PDF file, 2.3 MB. Copyright © 2022 Ginnan et al. 2022 Ginnan et al. https://creativecommons.org/licenses/by/4.0/ This content is distributed under the terms of the Creative Commons Attribution 4.0 International license.

A phylogenetic analysis of the core genera indicates that both bacterial and fungal root communities were rich in highly stable and phylogenetically diverse core taxa (Fig. S4). Root core genera from the bacterial clade *Alphaproteobacteria* (class) and the fungal family *Pleosporomycetidae* were all or nearly all binned as highly stable, indicating that genera in these clades were consistently high in relative abundance across all phenological stages. Medium- and low-stability core genera appear randomly dispersed across the root community phylogeny, with no obvious patterns.

However, leaf bacterial and fungal core community phylogenetic trees contained high, medium, and low stability patterns at the class and phylum levels (Fig. 4a and b). All core genera in the fungal class Tremellomycetes had medium to high stability. In contrast, all core genera in the fungal class Sordariomycetes had low stability across phenophases and met the defined core cutoffs only during fruit set or mature fruit stages. The leaf taxa within the bacterial class *Gammaproteobacteria* consisted of genera with high, medium, and low stability across the phenophases. Interestingly, all the *Gammaproteobacteria* were core members of the full flowering or floral bud development microbiomes regardless of their stability in other phenophases. Another distinct phylogenetic pattern observed in the leaf community was genera in the bacterial phylum *Actinobacteria* that had low or medium stability across all phenophases. However, 95.0% of core genera in the *Actinobacteria* clade were core during fruit set and/or fruit development. The only exception to this within the *Actinobacteria* clade was *Bifidobacterium*, which was associated only with full flowering and was not a core member of fruit set or fruit development microbiomes (Fig. 4a). Lastly, the leaf bacterial class *Betaproteobacteria* contains low- to medium-stability core genera with the most dispersed stage associations.

Overall, these data indicate that root bacterial and fungal communities have greater stability across phenophases than those of leaves (Fig. 4c and d). Additionally, core taxa had phylogenetically related trends within the high-, medium-, and low-stability classifications, indicating that conserved, vertically descended microbial traits may play a role in determining bacterial and fungal associations across phenophases, particularly in aboveground leaf tissue.

### Specific taxa were enriched in the foliar microbiome across the flowering phenophases.

**FIG 5:**
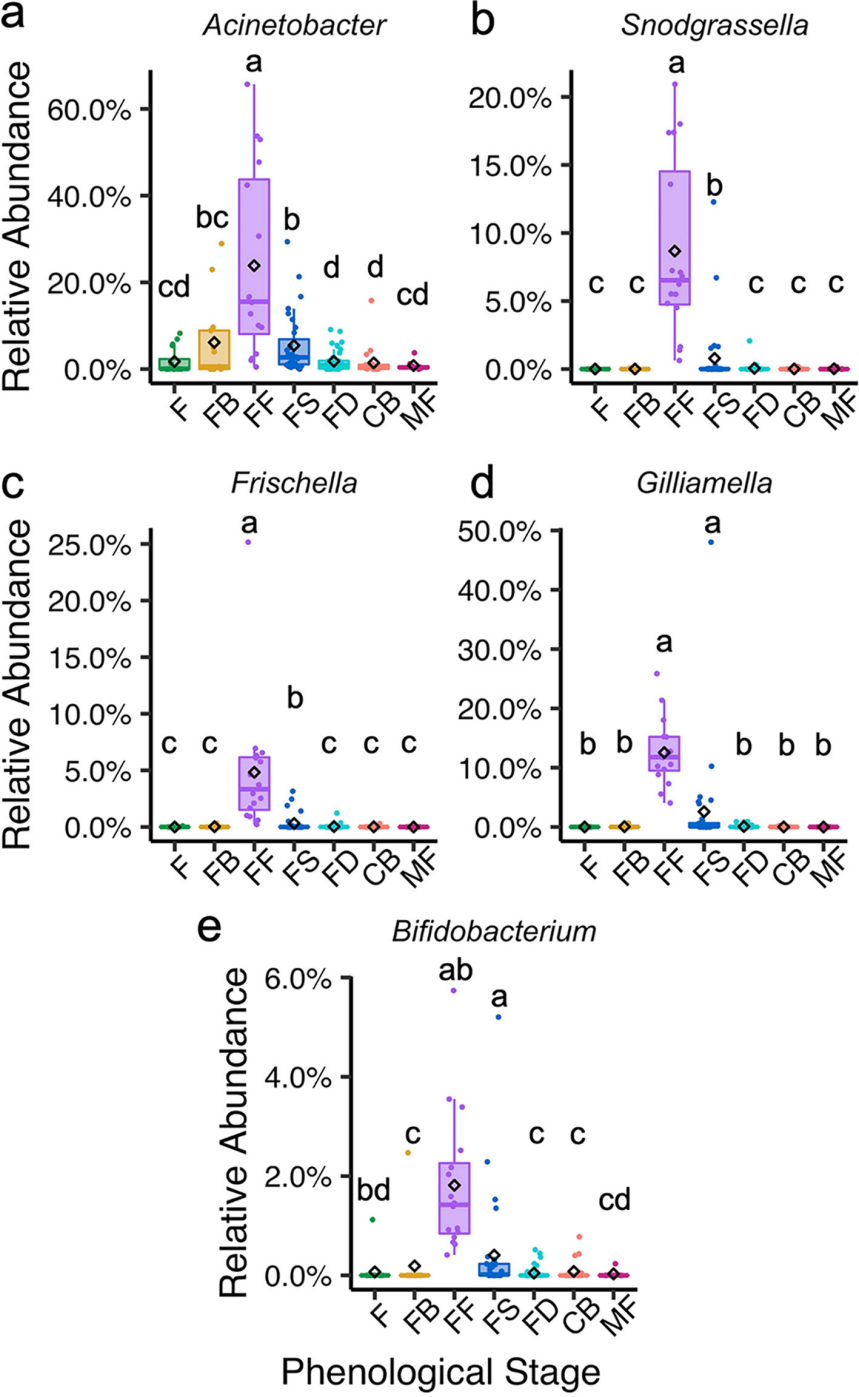
Core leaf bacteriome members enriched during full flowering. Gradually (a) and suddenly (b to e) enriched core (≥0.01% relative abundance and ≥75% prevalence) taxa during full flowering. The diamond symbol indicates the mean relative abundance. Letters indicate significant differences of *P ≤ *0.05, determined using DESeq2 GLM, Wald test with FDR adjustment. Phenological stages on the *x* axis include flush (F), floral bud break (FB), full flowering (FF), fruit set (FS), fruit development (FD), color break (CB), and mature fruit (MF).

We completed a genus-level differential relative abundance analysis on our list of core taxa that were ≥0.01% relative abundance and ≥75% prevalence in one or more phenophases. Our differential relative abundance analysis can determine finer-scale phenophase associations beyond just classification as a core microbiome member by looking for increases in relative abundance, proportionate to other members of the microbial community (enrichments), during specific phenophases. Ecologically dominant taxa (taxa with high abundance relative to that of other species in the community) are predicted to have a proportionately larger contribution to community function. Among all the phenophases, those associated with flowering (floral bud development and full flowering) had striking microbial enrichments, particularly among the leaf bacteria. *Acinetobacter* was a core member of five phenophases but was significantly enriched during full flowering compared to other phenophases (Fig. 5a). *Acinetobacter* had a gradual enrichment from flush and floral bud development to full flowering. This gradual enrichment signature indicates that *Acinetobacter* was present throughout the year but has a high temporal turnover rate that is in sync with the transitions from flush to floral bud development and then to full flowering.

We also observed bacteria that were sharply enriched during full flowering rather than undergoing gradual enrichments over the phenophases that lead up to full flowering (flush and floral bud development). These include *Snodgrassella*, *Frischella*, *Gilliamella*, and *Bifidobacterium* (Fig. 5b to e). The sharp enrichment patterns during full flowering suggest that these taxa were introduced into the community via a dispersal event.

### Foliar microbial depletions associated with flowering.

**FIG 6:**
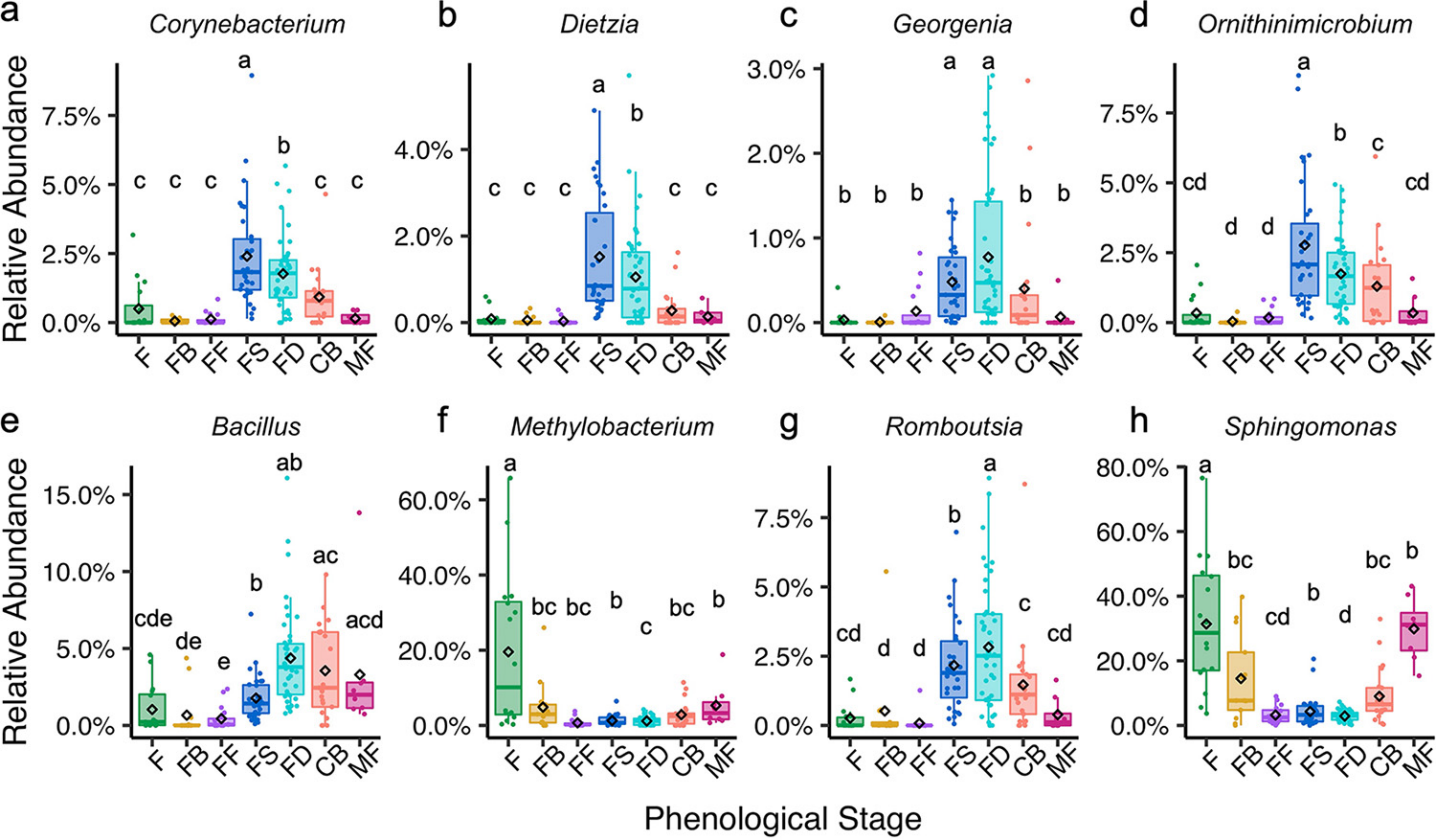
Core leaf bacteriome members depleted during full flowering. The diamond symbol indicates the mean relative abundance of core (≥0.01% relative abundance and ≥75% prevalence) taxa across stages. Letters indicate significant differences of *P ≤ *0.05, determined using DESeq2 GLM, Wald test with FDR adjustment. Phenological stages on the *x* axis include flush (F), floral bud break (FB), full flowering (FF), fruit set (FS), fruit development (FD), color break (CB), and mature fruit (MF).

We also identified bacterial leaf genera that had significant depletions (reduction in abundance relative to that of other taxa) during floral bud development and/or full flowering (Fig. 6a to h). Four *Actinobacter* genera, *Corynebacterium*, *Dietzia*, *Georgenia*, and *Ornithinimicrobium*, were significantly depleted during floral bud development and full flowering (Fig. 6a to d). *Bacillus*, *Methylobacterium*, *Romboutsia*, and *Sphingomonas* also significantly decreased in relative abundance during floral bud development and/or full flowering (Fig. 6e to h). For all differentially abundant genera, including bacteria and fungi, across all phenophases, see Fig. S5 and Table S4.

10.1128/mbio.00343-22.4 TABLE S4 All significant differentially abundant bacterial and fungal genera across phenological stages and tissue types. Download Table S4, XLSX file, 0.2 MB. Copyright © 2022 Ginnan et al. 2022 Ginnan et al. https://creativecommons.org/licenses/by/4.0/ This content is distributed under the terms of the Creative Commons Attribution 4.0 International license.

10.1128/mbio.00343-22.9 FIG S5 Core leaf mycobiome members with significant enrichments. The diamond symbol represents the mean relative abundance of core (≥0.01% relative abundance and ≥75% prevalence) taxa across stages. Letters indicate a significant difference of *P ≤ *0.05, determined using DESeq2 GLM, Wald test with FDR adjustment. Phenological stages on the *x* axis include flush (F), floral bud break (FB), full flowering (FF), fruit set (FS), fruit development (FD), color break (CB), and mature fruit (MF). Download FIG S5, TIF file, 1.3 MB. Copyright © 2022 Ginnan et al. 2022 Ginnan et al. https://creativecommons.org/licenses/by/4.0/ This content is distributed under the terms of the Creative Commons Attribution 4.0 International license.

### Microbe-microbe interactions contribute to phenophase-specific community structure.

**FIG 7:**
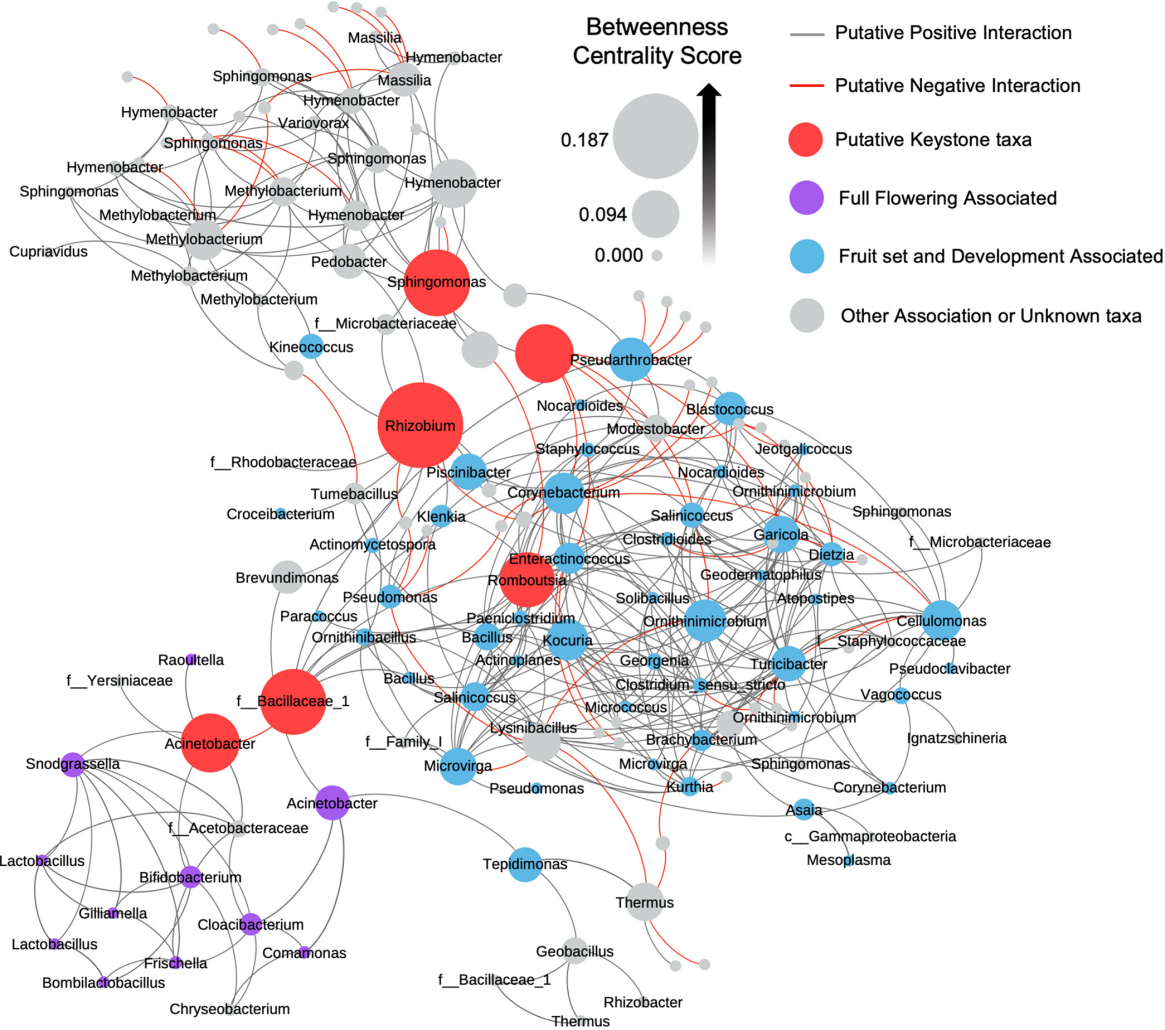
Leaf bacteriome interaction network. Each node represents a leaf bacteria OTU and is labeled with the lowest known taxonomic rank. Nodes are sized by betweenness centrality scores calculated using Gephi. Red nodes are predicted keystone taxa. Purple nodes are taxa significantly enriched during full flowering (FF), blue nodes were taxa significantly enriched during fruit set (FS) and/or fruit development (FD), and gray nodes are not significantly enriched during FF, FS, or FD. Lines represent predicted positive (black) and negative (red) interactions, determined using SPIEC-EASI sparse neighborhood covariance selection to infer interactions.

We performed a network analysis on the foliar bacterial communities from all samples. We focused on significantly enriched and/or depleted populations and populations with direct connections or putative first-degree interactions (neighbors). The goal of this approach was to give a broad overview of bacterial interactions across phenophases and identify taxa that potentially interact with specific phenophase-enriched taxa and potentially play a role in observed seasonal community compositional shifts. *Rhizobium*, *Sphingomonas*, an unknown bacterium, an unknown *Bacillaceae* (family), *Acinetobacter*, and *Romboutsia* have the highest normalized betweenness centrality scores ranging from 0.110 to 0.187. Betweenness centrality is a proxy for influence within a network because it measures how often a particular node (i.e., taxon) is the shortest connection or bridge between two other nodes. These high betweenness centrality scores and placement within the network indicate that these genera are potentially keystone taxa that may perform a stabilizing role in the microbial communities across phenological transitions and events (Fig. 7, red nodes). Groups of taxa connected by putative positive interactions cluster together to form distinct modules. These modules are separated by putative negative interactions. Our analysis organized bacterial taxa that were enriched in fruit set and fruit development into a single highly connective community module (cluster) (Fig. 7, blue nodes). This suggests that fruit set- and fruit development-associated microbiomes are compositionally similar and few microbe-microbe interactions change during the transition from fruit set to fruit development. Leaf bacteria associated with flowering also formed a module within the network (Fig. 7, purple nodes). Specific bacteria within the fruit set/development and flowering modules also interact with taxa that were enriched in the other four phenophases, which cluster together into a third module (Fig. 7, gray nodes). Overall, these predicted positive interactions represent inter- or codependent microbe-microbe relationships, and the putative negative interactions indicate potential direct (e.g., antibiosis) or indirect (e.g., resource exclusion) competition. These predicted microbe-microbe interactions within the microbiome likely affect community composition in addition to the exogenous influences of abiotic environmental conditions and biotic host physiological factors (e.g., carbon availability).

## DISCUSSION

The majority of studies examining how plant developmental stage affects the plant’s microbiome have focused on bacteria associated with the rhizosphere of annuals or herbaceous perennials such as maize (37, 38), rice (28), sorghum (27, 32), wheat (39), *Arabidopsis* (29), and *Boechera* (30). These important studies indicate that rhizosphere-associated microbiomes can shift in association with plant developmental stages in both domesticated and wild plants that have short-lived aboveground tissues. Studies of the endophytic xylem sap microbiome in grapevine, a deciduous perennial, also showed that microbial shifts were linked to changes in phenological stage (40). However, much less is known about how overall plant phenology affects above- and belowground microbiomes of evergreen woody perennials that have lifespans that can be decades long and can retain their leaves for multiple years, compared to annuals or deciduous perennials that produce and shed all their leaves each season. Here, we investigated microbiome compositional dynamics in above- and belowground tissues of mature 20-year-old *Citrus sinensis* trees to determine if temporal microbiome fluctuations were associated with host phenological events. The unique contribution of our research was the separation of leaf development from tree phenology. We did this by analyzing the changes in the foliar microbiome on fully mature leaves, which developed in the leaf cohort from the previous year, in relation to the phenological stages of the current year. Thus, the leaves were exposed to the same starting inoculum, minimizing the bias of any potential priority effects (i.e., order of arrival).

Our results indicate that the phyllosphere microbiome has an active and dynamic relationship with host phenology. More specifically, microbial shifts occurred as trees transitioned from the spring leaf flushing stage and entered flowering. The transition from spring flush to floral bud development and full flowering aligns with important transitions in source-to-sink transport of photosynthate in the tree (4). During foliar flushing periods, young leaves are a primary carbohydrate sink as they rapidly expand and mature. This source-to-sink transport of photosynthate shifts during floral bud break and development, when mature leaves transition to serve as source tissues to developing floral tissues that are also primary sink tissues. In addition to changes in source-to-sink transport, there are also significant changes in water dynamics within the canopy of the tree associated with full flowering. Flowers have the highest transpiration rate of the tree even compared to the leaves, which drastically increases the amount of water being transported into the overall canopy of the tree (4). Interestingly, the significant shift in overall foliar community composition from flushing to full flowering was not coupled with a change in species richness, indicating that the same taxa were present, just in different relative abundances in relation to one another. This demonstrates that foliar microbiome assemblage is changing in sync with tree physiology and development.

Empirical data, including presence/absence and relative abundance, can also be used to infer patterns or microbial enrichments and/or depletions relative to other taxa in the community, as well as ecological mechanisms that contribute to plant microbiome assembly, such as microbial species turnover and dispersal (41–43). Interestingly, microbial enrichment and depletion patterns of specific taxa suggest that microbial species turnover and dispersal events within the citrus microbiome occur in sync with phenological stage transitions. These enrichment/depletion patterns for specific taxa were more apparent in leaves than in the root compartment. Specifically, the bacterial genus *Acinetobacter* was enriched in leaves as trees transitioned from spring flush to floral bud development and peaked in relative abundance during full flowering, which is when mature leaves shift toward becoming source tissues for developing flowers and fruit. This may create a microenvironment that selects for an increase in relative abundance of these taxa when carbohydrate is translocating out of the leaves. Plant-associated *Acinetobacter* spp. have plant growth-promoting properties that include antagonism toward fungi (44) and the ability to solubilize phosphate and produce the plant hormone gibberellic acid (45, 46). *Acinetobacter* spp. are highly abundant in the floral nectar microbiome of *Citrus paradisi* and other plant species (47, 48) and were identified as a core member of the grapevine xylem sap microbiome (40). Their significant increase in relative abundance in the leaf microbiome at the time of flowering in citrus suggests a potential synergy between the foliar and floral microbiomes. *Acinetobacter* was also predicted to be a keystone taxon and was a major link between the flowering community and fruit set/development community clusters in our network. This enrichment in *Acinetobacter* may be simply due to selection imposed on the microbial community by the local plant environment, but it is tempting to speculate that *Acinetobacter* spp. provide an exogenous service to the plant by producing gibberellic acid and biologically available phosphorus to promote flowering that is in phase with its host’s phenological development. This hypothesis that the plant environment selects for taxa within its foliar microbiome that, in turn, promote its own reproductive growth warrants future inquiry. Specific bacterial enrichments also occurred at bloom time in grapevine, further supporting the speculation of a microbial role in plant growth development that is in sync with plant developmental stage (40).

We also observed signatures that indicate that specific taxa were depleted in relative abundance during flowering but enriched during fruit set. Phylogenetic reconstruction of these taxa indicated that the majority of the taxa belonging to the *Actinobacteria* phylum (19 of the 20 genera) were significantly depleted during flowering but subsequently enriched when trees begun to set fruit. This phylogenetic conservation of depletion/enrichment patterns within the *Actinobacteria* clade indicates that this is a nonrandom fluctuation within the microbiome structure associated with the transition from flowering to fruit production. As citrus trees set fruit, the fruits themselves begin exporting and importing hormones, such as indole acetic acid (IAA) and cytokinins, respectively (3). This results in a change in hormone levels in leaves as well. Phytohormones can affect microbiome composition by being directly utilized as a carbon source or through other undefined mechanisms (49, 50). We speculate that these hormonal shifts may place selective pressure on the foliar microbial community and lead to significant enrichments of *Actinobacteria* during fruit set and development. Specific differentially abundant taxa within the *Actinobacteria* clade that followed this pattern included *Corynebacterium*, *Dietzia*, *Georgenia*, and *Ornithinimicrobium*. Members of these genera can fix nitrogen and produce IAA, both of which are important supporters of fruit development (51–54). Thus, it is tempting to speculate that these taxa could play a role in coregulating fruit development in a manner that is synergistic with the host’s production of reproductive hormones. The biological role of *Actinobacteria* in the foliar microbiomes of plants is not well understood, but overall species richness was conserved across all phenological stages, except for fruit set, indicating that this phenological stage allows for microbial enrichments of specific taxa, particularly those belonging to the *Actinobacteria* phylum.

Genera outside the *Actinobacteria* clade were also depleted in leaves during floral bud development and full flowering, including *Bacillus*, *Methylobacterium*, *Romboutsia*, and *Sphingomonas*. Notably, *Romboutsia* and *Sphingomonas* were predicted to be keystone taxa in our microbe-microbe interaction network analysis, and all are in the top 20% highest betweenness centrality scores. Keystone taxa play a stabilizing role in microbial communities. Depletion of these taxa during flowering may have cascading effects that influence microbial species turnover by allowing other taxa, such as the *Actinobacteria*, to flourish during subsequent developmental stages, like fruit set. This suggests that microbial turnover in the foliar microbiome is mediated by selective pressures imposed by the plant developmental stage in conjunction with microbe-microbe interactions to modulate community diversity and composition. Further experimentation is needed to confirm these interactions.

Microbial dispersal events can drive microbial turnover and influence the relative abundance of endogenous taxa in the community. Full flowering is a dynamic phenophase in plant development where there are frequent interactions between plants and pollinator species that rely on floral resources, like nectar and pollen. These macro-level interactions can also have effects at the microorganismal level. Pollinator (e.g., bee) visitation alters flower surface, nectar, and subsequent seed microbial community composition (55–58). During flowering, we observed striking microbial enrichments of bacteria taxa belonging to the *Betaproteobacteria* and *Gammaproteobacteria* clades that include *Gilliamella*, *Snodgrassella*, *Bifidobacterium*, and *Frischella.* These enrichments in these anaerobic taxa were unique to the flowering phenophase and quickly declined following flowering, suggesting they are immigrants to the community and not endogenous members of the native microbiome. Moreover, these anaerobic taxa are prevalent in the bee gut microbiome (59). We hypothesize that these taxa immigrated into the citrus microbiome via a dispersal event during bee visitation. We consider this external influence host phenology-associated because phenophase-specific plant morphology (i.e., flowers) regulate this diffuse interaction. Bacteria can be introduced to plants by bees and potentially migrate from the flower to the vascular bundles resulting in systemic movement within the plant (60–62). Leaf carbohydrate content is highest during flowering, which may promote the growth of these fermenting bacteria (4). Notably, *Bifidobacterium* was the only core leaf genus from the *Actinobacteria* phylum that was enriched during flowering, whereas the other 19 *Actinobacteria* taxa were depleted during flowering. This further supports the hypothesis that *Bifidobacterium* was introduced via a dispersal event and is not part of the endogenous microbiota like the other taxa in the plant-associated *Actinobacteria* clade. Nectar-inhabiting bacteria can influence nectar volatile profiles that, in turn, influence pollinator visitation preferences (63), and it would be interesting to determine if these putative immigrants contribute to shifts in nectar volatile profiles that affect bee feeding behaviors.

The next frontier in microbiome research is to determine the functional roles that microbes play in microbe-microbe and host-microbiome interactions. Martiny et al. found that conservation of microbial traits was linked more strongly to vertical phylogenetic relatedness of the microorganisms within a microbiome than to traits that are shared among taxa by horizontal gene transfer (64). Similarly, we also observed phylogenetic conservation within microbial enrichments, suggesting that those groups of related organisms play similar functional roles during specific phenophases or across several phenophases. We speculate that taxa with high stability across phenophases may serve a community-stabilizing function, while low stability or phenophase-specific core microbes likely have more specialized, transient roles in the community. Because microbes can alter host phenology (18, 21, 65–68), which is a critical factor in plant health and productivity, incorporation of microbial presence/absence and patterns of enrichment into plant phenological models may improve phenophase timing predictions once the functional roles of these microbes are determined. This information could also lead to the commercialization of biofertilizers for horticultural purposes that could be applied at specific plant life stages to enhance crop productivity.

## MATERIALS AND METHODS

### Field site and study design.

Our longitudinal study included eight Late Navel Powell sweet orange trees (*Citrus sinensis* L. Osbeck, cv Powell) on Carrizo rootstock grown at the University of California (UC) Lindcove Research and Extension Center (LREC) in Exeter, CA. Trees were planted in 1997 (20 to 22 years old at time of sampling) and managed with conventional farming strategies similar to those of industry orchards. Sampling occurred at 20 time points (monthly for first sample year, targeted sampling for second year) from July 2017 to April 2019. Prior to each monthly sampling, tagged trees were visually assessed and developmental stages were recorded. We recorded the dominant stage occurring during each sampling event, but note that phenophases can overlap, particularly during transitions. We avoided sampling during transitions between stages when possible. Seven major phenological stages were used for categorization in this study. These included (i) spring vegetative shoot flush (soft “feather” flush, early elongation and leaf emission [phase 2: stages V2 to V3; see reference 69]), referred to as “flush” (F; February to March), (ii) early floral bud break and development, referred to as “floral bud development” (FB; March), (iii) full flowering (FF; April), (iv) fruit set (FS; May to July), (v) exponential fruit growth and development, referred to as “fruit development” (FD; August to October), (vi) color break (CB; initiation of fruit maturation; November to December), and (vii) mature fruit (MF; January) (Fig. 1) (70). In California, sweet orange trees can have foliar flushing events in the spring, summer, and fall. However, spring flush events are robust and synchronized among trees, whereas summer and fall flushes are more sporadic and weaker. Therefore, we focused on the spring vegetative flush for this study and sampled when all parts of the canopy for all trees had new flush. At the time of all other sampling events, there was only 0 to 10% new flush intermittently dispersed across trees and individual canopies. Citrus root flushes are known to occur in June and November in California but are difficult to observe in large field trees because they are under the soil; therefore, the exact timing of root flush events was not included as metadata in this study. Mature fruits were harvested between our April FF and May FS sampling events. Fertilizer treatments and/or amendments and number of hours irrigated were collected as monthly metadata variables.

### Leaf and root sampling.

Leaves sampled were fully mature, with a fully hardened cuticle, and dark green (no yellowing or signs of senescence). Leaves were from one of the previous year’s flush events and, thus, in a developmental cohort separate from that of the reproductive structures on the trees at the time of sampling. During sampling, each tree was divided into 4 quadrants (north, south, east, and west), and two stems with at least two leaves of the target age attached (petiole attached) were collected from each quadrant with sterilized hand clippers and pooled into a sterile 24-oz. standup whirl-pak bag (Nasco B01401, Fort Atkinson, WI). Both fruit-bearing and nonbearing branches were collected at random, and all stems/leaves from a single tree were pooled and immediately placed on ice in a cooler for transit to the laboratory.

Fibrous or feeder roots were sampled from two sides of the tree approximately 0.5 m away from the base of the trunk near the irrigation line. A small hand shovel was used to remove the topsoil until roots were visible. Approximately 5 g of fibrous roots were gently pulled from the ground and shaken until all loose soil was removed. Then, roots were sealed in a sterile 24-oz. standup whirl-pak bag (Nasco B01401, Fort Atkinson, WI) and immediately placed on ice in a cooler for transit to the laboratory.

Gloves were changed and clippers and hand shovels were sterilized with 30% household bleach between trees. All samples were transferred to the LREC onsite laboratory where they were immediately frozen at −20°C. Samples were inspected by the California Department of Food and Agriculture according to California citrus quarantine protocols prior to overnight shipment to UC Riverside on dry ice.

### Tissue processing and DNA extractions.

At UC Riverside, samples were kept frozen on dry ice during processing for downstream DNA extractions. For leaf samples,1 to 2 leaves were removed from each of the eight stems in the pooled sample bag (total of 8 to 10 leaves per tree sample). Leaf tissue was chopped into 1- to 2-cm pieces and put in 50-mL conical tubes (Falcon 352098, Corning, Glendale, AZ). Roots were rinsed with sterile water to remove surface soil and placed in a 50-mL conical tube (Falcon 352098, Corning, Glendale, AZ). All leaf and root samples were stored at −80°C and then lyophilized with a benchtop freeze dryer (Labconco FreeZone 4.5L, Kansas City, MO) for 16 to 20 h. Dried samples were crushed into <0.5-cm fragments and homogenized in the 50-mL conical tubes using a flame-sterilized metal spatula. Leaf and root DNA extractions were performed according to published protocols (22). Briefly, 100 mg of freeze-dried crushed tissue was transferred to 2-mL microcentrifuge tubes (Eppendorf Safe-Lock tubes; Eppendorf, Hamburg, Germany) containing a 4-mm stainless-steel grinding ball (SPEX SamplePrep, Metuchen, NJ, USA). Samples were chilled at −80°C for 15 min and then pulverized into a powder using a 2010 Geno/Grinder (SPEX SamplePrep) at 1,680 rpm for 20 s (×2). Then, 1 mL of 4 M guanidine thiocyanate was added to the pulverized leaf and root samples. All samples were incubated at 4°C for 15 min. Next, samples were centrifuged for 1 h at 17,500 × *g*. DNA was isolated from the supernatant using the MagMAX-96 DNA multi-sample kit (Thermo Fisher Scientific) on a MagMAX Express-96 deep well magnetic particle processor. DNA was eluted in 100 mL of DNA elution buffer and stored at −20°C until utilized for bacterial and fungal Illumina library construction.

### High-throughput sequencing library preparation.

Leaf and root bacterial communities were sequenced from all leaf (*n* = 159) and root (*n* = 159) samples (20 time points). Bacterial Illumina Miseq libraries were built by amplifying the bacterial rRNA 16S V4 region using the 515FB/806RB primer set and the standardized Earth Microbiome Project protocol, which can be found online (https://earthmicrobiome.org/protocols-and-standards/16s/) (71). To limit the amplification of plant mitochondrial and plastid 16S regions, we used pPNA and mPNA clamps (PNA Bio, Newbury Park, CA), which bind to these plant sequences and block binding of the bacterial 515FB/806RB (72, 73). Leaf sample reactions received 0.75 μM pPNA and mPNA clamps, and 0.75 μM mPNA was added to each root sample reaction.

The first 14 consecutive monthly leaf and root samples were included in fungal community libraries. Our preliminary analyses shifted our main focus to bacterial communities, and therefore we did not sequence the fungal communities of leaf/roots collected at the last 6 time points in the second sampling year. Fungal Illumina MiSeq libraries were built by amplifying the fungal ITS1 region using the ITS1f/ITS2 primer set and the standardized Earth Microbiome Project protocol.

Triplicate PCRs for each sample were pooled, and amplification was verified on a 1% agarose gel. Amplicon samples were quantified for DNA concentration using Quant-iT PicoGreen (Invitrogen). Equal amounts of amplicons (240 ng) from each sample were pooled and AMPure XP (Beckman Coulter) beads were used to clean the sample library. The cleaned library was then quantified using Qubit (Qiagen; 260/280). The libraries were diluted to 20 to 30 μg/mL, and a final quality assessment was done with a Bioanalyzer at the UCR Genomics Core facility. Paired-end sequencing (2 × 300) was performed on an Illumina MiSeq platform with a 20% PhiX spike included before sequencing.

### Data processing and statistics.

Demultiplexed, PhiX reads-removed, and Illumina adapter-trimmed sequences were received from the UCR Genomics Core. Amplicon sequencing raw reads for 16S rRNA genes and fungal ITS2 region are available on the NCBI SRA database under BioProject accession number PRJNA685913. Bacterial and fungal reads were preprocessed using a USEARCH (v9.1.13)/VSEARCH (v2.3.2) pipeline. Forward and reverse sequencing files were joined with USEARCH allowing for staggered ends and up to 10 mismatches. After quality filtering using VSEARCH, there were 65.5 million 16S reads and 21.1 million fungal reads. Sequences were dereplicated, singletons were removed, and OTUs were formed using USEARCH with a 97% similarity cutoff. The bacterial library produced 19,626 OTUs, which were assigned to 4,183 taxonomic names using the RDP 16S database, v18. The fungal library produced 44,447 OTUs, which were assigned to 31,056 taxonomic names using the UNITE fungal reference database, v02.02.2019. On average, 12.7% of the 16S library reads from each leaf and root sample were assigned as bacteria. See Table S1 in the supplemental material for all 16S bacterial read counts of individual samples. The remaining sequences, which were removed, were attributed to chloroplast, plant mitochondria, or *Archaea* or could not be assigned to a kingdom. Our fungal libraries did not have nonspecific binding issues. USEARCH was also used to create phylogenetic tree files in Newick format.

10.1128/mbio.00343-22.1 TABLE S1 Bacterial 16S read counts. Download Table S1, XLSX file, 0.02 MB. Copyright © 2022 Ginnan et al. 2022 Ginnan et al. https://creativecommons.org/licenses/by/4.0/ This content is distributed under the terms of the Creative Commons Attribution 4.0 International license.

Preprocessed taxonomically assigned OTU tables were imported into R (v3.6.0). Samples with fewer than 1,000 reads were removed. Reads were rarified to even depth for each alpha diversity comparison. Alpha diversity was compared using the number of OTUs observed (74). A ranked sums analysis of variance statistical test, Kruskal-Wallis, followed by a pairwise Dunn’s test with Holm’s correction for multiple comparisons, was used to calculate *P* values.

Beta diversity analyses were performed using R packages phyloseq (v1.28.0) and vegan (v2.5.6) (74, 75). Preprocessed reads were transformed using total sum scaling normalization. Using the ordinate() and plot_ordination() functions, a principal coordinate analysis (PCoA) was done on weighted Unifrac distances, which accounts for relative relatedness and quantitative variance of communities. Ninety-five percent confidence ellipses were added to further examine groups using the stat_ellipse() function. A permutational multivariate analysis of variance (PERMANOVA) statistical test was performed on weighted Unifrac distances that were square-root transformed using the vegan::adonis2() function, including the following covariates and interaction terms in the model: adonis2(dist.matrix ~ phenology*Sample_year + Fertilizer + Mean_Temp*hrs_irrigated + Total_rain, permutations = 999, method = “wunifrac,” sqrt.dist = TRUE). Pairwise PERMANOVA with false-discovery rate (FDR) correction was accomplished with RVAideMemoire::pairwise.perm.manova() (v0.9.74) (76). Core microbiota identifications were performed using microbiome::core() (v1.6.0) with prevalence set at 0.75 and detection set at 0.01/100 (77). The core root bacteriome was also defined with prevalence set at 0.75 and detection set at 0.1/100, which did not affect the interpretation of the results but greatly improved readability of the phylogenetic tree.

To understand if phylogenetic relationships affect microbial associations with phenological stages, core genera taxonomic assignments were input into a phylogenetic tree generator, phyloT v2 (https://phylot.biobyte.de/index.cgi), to generate a Newick format phylogenetic tree. Tree and metadata were visualized using the interactive tree of life visualization program, iTOL (https://itol.embl.de/) (78). Genera with fewer than 50 reads were filtered out, and differentially abundant populations at the genus level were identified using DESeq2 (v1.24.0) to run a parametric fit for dispersion on a negative binomial generalized linear model, followed by a Wald test with FDR adjustment to produce *P* values (79). Input root and leaf bacterial OTU tables used in DESeq2 analysis were rescaled using a pseudocount of 1. All possible phenological stage pairwise combinations were tested using results() and the contrast option. Using ggplot2 (v3.2.1) and phyloseq::subset_taxa() function, the relative abundance of specific species were plotted as boxplots (74, 80).

The top 300 most abundant leaf bacterial OTUs were input into a network analysis using Sparse Inverse Covariance Estimation for Ecological Association Inference, Spiec-Easi (v1.0.7), which infers interactions using neighborhood selection and the concept of conditional independence, rather than a standard correlation or covariance estimation (81). With set.seed(1244), neighborhood modeling (mb) was executed with the nlambda set to 70 and rep.num set at 99; standard settings were used for all other parameters. Spiec-Easi results were converted to igraph format and imported into Gephi (v0.9.2). The network was visualized using a Yifan Hu layout, and taxa were filtered to focus on differentially abundant taxa and immediate neighbors. Betweenness centralities were calculated using Gephi network diameter statistics, and centralities were normalized to a 0 to 1 scale. Taxa were further filtered by abundance, with the final figure showcasing the 155 most abundant OTUs within the above parameters. This significantly increased readability without affecting the interpretation of the results.

### Data availability.

The data that support the findings of this study are openly available in the NCBI SRA database under BioProject PRJNA685913.

## References

[1] Nord EA, Lynch JP. 2009. Plant phenology: a critical controller of soil resource acquisition. J Exp Bot 60:1927–1937. doi:10.1093/jxb/erp018. 19286918

[2] Wohlfahrt G, Tomelleri E, Hammerle A. 2019. The urban imprint on plant phenology. Nat Ecol Evol 3:1668–1674. doi:10.1038/s41559-019-1017-9. 31712692 PMC6882677

[3] McAtee P, Karim S, Schaffer R, David K. 2013. A dynamic interplay between phytohormones is required for fruit development, maturation, and ripening. Front Plant Sci 4:79. doi:10.3389/fpls.2013.00079. 23616786 PMC3628358

[4] Goldschmidt EE, Koch KE. 2017. Citrus, p 797–824. *In* Photoassimilate distribution plants and crops source-sink relationships. Routledge, Oxfordshire, UK.

[5] Rosbakh S, Poschlod P. 2016. Minimal temperature of pollen germination controls species distribution along a temperature gradient. Ann Bot 117:1111–1120. doi:10.1093/aob/mcw041. 27192710 PMC4904169

[6] Wolkovich EM, Burge DO, Walker MA, Nicholas KA. 2017. Phenological diversity provides opportunities for climate change adaptation in winegrapes. J Ecol 105:905–912. doi:10.1111/1365-2745.12786.

[7] Hegland SJ, Nielsen A, Lázaro A, Bjerknes A-L, Totland Ø. 2009. How does climate warming affect plant-pollinator interactions? Ecol Lett 12:184–195. doi:10.1111/j.1461-0248.2008.01269.x. 19049509

[8] Forrest JRK. 2015. Plant-pollinator interactions and phenological change: what can we learn about climate impacts from experiments and observations? Oikos 124:4–13. doi:10.1111/oik.01386.

[9] Kudo G, Ida TY. 2013. Early onset of spring increases the phenological mismatch between plants and pollinators. Ecology 94:2311–2320. doi:10.1890/12-2003.1. 24358716

[10] Baker RA. 1994. Potential dietary benefits of citrus pectin and fiber. Food Technol 48:133–139.

[11] Economos C, Clay WD. 1999. Nutritional and health benefits of citrus fruits. Energy 62:37.

[12] United States Department of Agriculture Foreign Agricultural Service. 2022. Citrus: world markets and trade. World Agricultural Outlook Board/USDA, Washington, DC.

[13] Albrigo LG, Beck HW, Valiente JI. 2006. Testing a flowering expert system for the ‘decision information system for citrus’. Acta Hortic 707:17–24. doi:10.17660/ActaHortic.2006.707.1.

[14] Peres NAR, Souza NL, Furtado EL, Timmer LW. 2004. Evaluation of systems for timing of fungicide sprays for control of postbloom fruit drop of citrus in Brazil. Plant Dis 88:731–735. doi:10.1094/PDIS.2004.88.7.731. 30812484

[15] Bellows TS, Morse JG, Lovatt CJ. 1989. Modelling flower development in citrus. Manipulation of Fruiting 115–129. doi:10.1016/b978-0-408-02608-6.50014-x.

[16] Mechlia NB, Carroll JJ. 1989. Agroclimatic modeling for the simulation of phenology, yield and quality of crop production. Int J Biometeorol 33:36–51. doi:10.1007/BF01045896.

[17] Wagner MR, Lundberg DS, Coleman-Derr D, Tringe SG, Dangl JL, Mitchell-Olds T. 2014. Natural soil microbes alter flowering phenology and the intensity of selection on flowering time in a wild Arabidopsis relative. Ecol Lett 17:717–726. doi:10.1111/ele.12276. 24698177 PMC4048358

[18] Lu T, Ke M, Lavoie M, Jin Y, Fan X, Zhang Z, Fu Z, Sun L, Gillings M, Peñuelas J, Qian H, Zhu Y-G. 2018. Rhizosphere microorganisms can influence the timing of plant flowering. Microbiome 6:231. doi:10.1186/s40168-018-0615-0. 30587246 PMC6307273

[19] Lau JA, Lennon JT. 2011. Evolutionary ecology of plant–microbe interactions: soil microbial structure alters selection on plant traits. New Phytol 192:215–224. doi:10.1111/j.1469-8137.2011.03790.x. 21658184

[20] Lau JA, Lennon JT. 2012. Rapid responses of soil microorganisms improve plant fitness in novel environments. Proc Natl Acad Sci USA 109:14058–14062. doi:10.1073/pnas.1202319109. 22891306 PMC3435152

[21] O’Brien A, Ginnan N, Rebolleda-Gomez M, Wagner MR. 2021. Microbial effects on plant phenology and fitness. EcoEvoRxiv. https://ecoevorxiv.org/exadg/. 10.1002/ajb2.1743 34655479

[22] Ginnan NA, Dang T, Bodaghi S, Ruegger PM, Peacock BB, McCollum G, England G, Vidalakis G, Roper C, Rolshausen P, Borneman J. 2018. Bacterial and fungal next generation sequencing datasets and metadata from citrus infected with ‘*Candidatus* Liberibacter asiaticus’. Phytobiomes 2:64–70. doi:10.1094/PBIOMES-08-17-0032-A.

[23] Blacutt A, Ginnan N, Dang T, Bodaghi S, Vidalakis G, Ruegger P, Peacock B, Viravathana P, Campos VF, Drozd C, Jablonska B, Borneman J, McCollum G, Cordoza J, Meloch J, Berry V, Salazar LL, Maloney KN, Rolshausen PE, Roper MC. 2020. An in vitro pipeline to screen and select citrus-associated microbiota with potential anti-*Candidatus* Liberibacter asiaticus properties. Appl Environ Microbiol 86. doi:10.1128/AEM.02883-19. PMC7117939 32086307

[24] Riera N, Handique U, Zhang Y, Dewdney MM, Wang N. 2017. Characterization of antimicrobial-producing beneficial bacteria isolated from Huanglongbing escape citrus trees. Front Microbiol 8:2415. doi:10.3389/fmicb.2017.02415. 29375487 PMC5770638

[25] Xu J, Zhang Y, Zhang P, Trivedi P, Riera N, Wang Y, Liu X, Fan G, Tang J, Coletta-Filho HD, Cubero J, Deng X, Ancona V, Lu Z, Zhong B, Roper MC, Capote N, Catara V, Pietersen G, Vernière C, Al-Sadi AM, Li L, Yang F, Xu X, Wang J, Yang H, Jin T, Wang N. 2018. The structure and function of the global citrus rhizosphere microbiome. Nat Commun 9:4894. doi:10.1038/s41467-018-07343-2. 30459421 PMC6244077

[26] Ginnan NA, Dang T, Bodaghi S, Ruegger PM, McCollum G, England G, Vidalakis G, Borneman J, Rolshausen PE, Roper MC. 2020. Disease-induced microbial shifts in citrus indicate microbiome-derived responses to Huanglongbing across the disease severity spectrum. Phytobiomes J 4:375–387. doi:10.1094/PBIOMES-04-20-0027-R.

[27] Schlemper TR, Leite MFA, Lucheta AR, Shimels M, Bouwmeester HJ, van Veen JA, Kuramae EE. 2017. Rhizobacterial community structure differences among sorghum cultivars in different growth stages and soils. FEMS Microbiol Ecol 93. doi:10.1093/femsec/fix096. 28830071

[28] Edwards JA, Santos-Medellín CM, Liechty ZS, Nguyen B, Lurie E, Eason S, Phillips G, Sundaresan V. 2018. Compositional shifts in root-associated bacterial and archaeal microbiota track the plant life cycle in field-grown rice. PLoS Biol 16:e2003862. doi:10.1371/journal.pbio.2003862. 29474469 PMC5841827

[29] Chaparro JM, Badri DV, Vivanco JM. 2014. Rhizosphere microbiome assemblage is affected by plant development. ISME J 8:790–803. doi:10.1038/ismej.2013.196. 24196324 PMC3960538

[30] Wagner MR, Lundberg DS, del Rio TG, Tringe SG, Dangl JL, Mitchell-Olds T. 2016. Host genotype and age shape the leaf and root microbiomes of a wild perennial plant. Nat Commun 7. doi:10.1038/ncomms12151. PMC4945892 27402057

[31] Xiong C, Singh BK, He J-Z, Han Y-L, Li P-P, Wan L-H, Meng G-Z, Liu S-Y, Wang J-T, Wu C-F, Ge A-H, Zhang L-M. 2021. Plant developmental stage drives the differentiation in ecological role of the maize microbiome. Microbiome 9:171. doi:10.1186/s40168-021-01118-6. 34389047 PMC8364065

[32] Xu L, Naylor D, Dong Z, Simmons T, Pierroz G, Hixson KK, Kim Y-M, Zink EM, Engbrecht KM, Wang Y, Gao C, DeGraaf S, Madera MA, Sievert JA, Hollingsworth J, Birdseye D, Scheller HV, Hutmacher R, Dahlberg J, Jansson C, Taylor JW, Lemaux PG, Coleman-Derr D. 2018. Drought delays development of the sorghum root microbiome and enriches for monoderm bacteria. Proc Natl Acad Sci USA 115:E4284–E4293. doi:10.1073/pnas.1717308115. 29666229 PMC5939072

[33] Dombrowski N, Schlaeppi K, Agler MT, Hacquard S, Kemen E, Garrido-Oter R, Wunder J, Coupland G, Schulze-Lefert P. 2017. Root microbiota dynamics of perennial *Arabis alpina* are dependent on soil residence time but independent of flowering time. ISME J 11:43–55. doi:10.1038/ismej.2016.109. 27482927 PMC5097464

[34] Dibner RR, Weaver AM, Brock MT, Custer GF, Morrison HG, Maignien L, Weinig C. 2021. Time outweighs the effect of host developmental stage on microbial community composition. FEMS Microbiol Ecol 97:fiab102. doi:10.1093/femsec/fiab102. 34259857

[35] Keutgen N, Chen KAI. 2001. Responses of citrus leaf photosynthesis, chlorophyll fluorescence, macronutrient and carbohydrate contents to elevated CO2. J Plant Physiol 158:1307–1316. doi:10.1078/0176-1617-00564.

[36] Rhoads WA, Wedding RT. 1953. Leaf drop in citrus: excessive fall regardless of cause may lower soluble solids in fruit. Calif Agric 7:9.

[37] Emmett BD, Buckley DH, Drinkwater LE. 2020. Plant growth rate and nitrogen uptake shape rhizosphere bacterial community composition and activity in an agricultural field. New Phytol 225:960–973. doi:10.1111/nph.16171. 31487394

[38] Aira M, Gómez-Brandón M, Lazcano C, Bååth E, Domínguez J. 2010. Plant genotype strongly modifies the structure and growth of maize rhizosphere microbial communities. Soil Biol Biochem 42:2276–2281. doi:10.1016/j.soilbio.2010.08.029.

[39] Gdanetz K, Trail F. 2017. The wheat microbiome under four management strategies, and potential for endophytes in disease protection. Phytobiomes J 1:158–168. doi:10.1094/PBIOMES-05-17-0023-R.

[40] Deyett E, Rolshausen PE. 2019. Temporal dynamics of the sap microbiome of grapevine under high Pierce’s disease pressure. Front Plant Sci 10:1246. doi:10.3389/fpls.2019.01246. 31681363 PMC6805966

[41] Kraft NJB, Ackerly DD. 2014. Assembly of plant communities. Ecology and the Environment 8:67–88.

[42] Cordovez V, Dini-Andreote F, Carrión VJ, Raaijmakers JM. 2019. Ecology and evolution of plant microbiomes. Annu Rev Microbiol 73:69–88. doi:10.1146/annurev-micro-090817-062524. 31091418

[43] Liang Y, Jiang Y, Wang F, Wen C, Deng Y, Xue K, Qin Y, Yang Y, Wu L, Zhou J, Sun B. 2015. Long-term soil transplant simulating climate change with latitude significantly alters microbial temporal turnover. ISME J 9:2561–2572. doi:10.1038/ismej.2015.78. 25989371 PMC4817637

[44] Liu CH, Chen X, Liu TT, Lian B, Gu Y, Caer V, Xue YR, Wang BT. 2007. Study of the antifungal activity of *Acinetobacter baumannii* LCH001 in vitro and identification of its antifungal components. Appl Microbiol Biotechnol 76:459–466. doi:10.1007/s00253-007-1010-0. 17534613

[45] Kang S-M, Khan AL, Hamayun M, Shinwari ZK, Kim Y-H, Joo G-J, Lee I-J. 2012. *Acinetobacter calcoaceticus* ameliorated plant growth and influenced gibberellins and functional biochemicals. Pak J Bot 44:365–372.

[46] Kang S-M, Joo G-J, Hamayun M, Na C-I, Shin D-H, Kim HY, Hong J-K, Lee I-J. 2009. Gibberellin production and phosphate solubilization by newly isolated strain of *Acinetobacter calcoaceticus* and its effect on plant growth. Biotechnol Lett 31:277–281. doi:10.1007/s10529-008-9867-2. 18931973

[47] Fridman S, Izhaki I, Gerchman Y, Halpern M. 2012. Bacterial communities in floral nectar. Environ Microbiol Rep 4:97–104. doi:10.1111/j.1758-2229.2011.00309.x. 23757235

[48] Álvarez-Pérez S, Herrera CM. 2013. Composition, richness and nonrandom assembly of culturable bacterial–microfungal communities in floral nectar of Mediterranean plants. FEMS Microbiol Ecol 83:685–699. doi:10.1111/1574-6941.12027. 23057414

[49] Lebeis SL, Paredes SH, Lundberg DS, Breakfield N, Gehring J, McDonald M, Malfatti S, Glavina del Rio T, Jones CD, Tringe SG, Dangl JL. 2015. Plant microbiome: salicylic acid modulates colonization of the root microbiome by specific bacterial taxa. Science 349:860–864. doi:10.1126/science.aaa8764. 26184915

[50] Carvalhais LC, Dennis PG, Badri DV, Kidd BN, Vivanco JM, Schenk PM. 2015. Linking jasmonic acid signaling, root exudates, and rhizosphere microbiomes. Mol Plant Microbe Interact 28:1049–1058. doi:10.1094/MPMI-01-15-0016-R. 26035128

[51] Sebastian J, Chandra AK, Kolattukudy PE. 1987. Discovery of a cutinase-producing *Pseudomonas* sp. cohabiting with an apparently nitrogen-fixing *Corynebacterium* sp. in the phyllosphere. J Bacteriol 169:131–136. doi:10.1128/jb.169.1.131-136.1987. 3793714 PMC211744

[52] Diba F, Sannyal SK, Alam SMS, Hossain MA, Sultana M. 2016. Plant growth promoting ability of soil arsenite resistant bacteria. Bangla J Microbiol 32:25–31. doi:10.3329/bjm.v32i0.28474.

[53] Huo Y, Kang JP, Ahn JC, Kim YJ, Piao CH, Yang DU, Yang DC. 2021. Siderophore-producing rhizobacteria reduce heavy metal-induced oxidative stress in *Panax ginseng* Meyer. J Ginseng Res 45:218–227. doi:10.1016/j.jgr.2019.12.008. 33841002 PMC8020345

[54] Rilling JI, Acuña JJ, Sadowsky MJ, Jorquera MA. 2018. Putative nitrogen-fixing bacteria associated with the rhizosphere and root endosphere of wheat plants grown in an Andisol from Southern Chile. Front Microbiol 9:2710. doi:10.3389/fmicb.2018.02710. 30524385 PMC6256256

[55] Tsuji K, Fukami T. 2018. Community‐wide consequences of sexual dimorphism: evidence from nectar microbes in dioecious plants. Ecology 99:2476–2484. doi:10.1002/ecy.2494. 30216955

[56] Ushio M, Yamasaki E, Takasu H, Nagano AJ, Fujinaga S, Honjo MN, Ikemoto M, Sakai S, Kudoh H. 2015. Microbial communities on flower surfaces act as signatures of pollinator visitation. Sci Rep 5:8695. doi:10.1038/srep08695. 25733079 PMC4346974

[57] Aizenberg-Gershtein Y, Izhaki I, Halpern M. 2013. Do honeybees shape the bacterial community composition in floral nectar? PLoS One 8:e67556. doi:10.1371/journal.pone.0067556. 23844027 PMC3701072

[58] Prado A, Marolleau B, Vaissière BE, Barret M, Torres-Cortes G. 2020. Insect pollination: an ecological process involved in the assembly of the seed microbiota. Sci Rep 10:3575. doi:10.1038/s41598-020-60591-5. 32107443 PMC7046713

[59] Powell JE, Martinson VG, Urban-Mead K, Moran NA. 2014. Routes of acquisition of the gut microbiota of the honey bee *Apis mellifera*. Appl Environ Microbiol 80:7378–7387. doi:10.1128/AEM.01861-14. 25239900 PMC4249178

[60] Kim D-R, Cho G, Jeon C-W, Weller DM, Thomashow LS, Paulitz TC, Kwak Y-S. 2019. A mutualistic interaction between *Streptomyces* bacteria, strawberry plants and pollinating bees. Nat Commun 10:4802. doi:10.1038/s41467-019-12785-3. 31641114 PMC6805876

[61] Cellini A, Giacomuzzi V, Donati I, Farneti B, Rodriguez-Estrada MT, Savioli S, Angeli S, Spinelli F. 2019. Pathogen-induced changes in floral scent may increase honeybee-mediated dispersal of *Erwinia amylovora*. ISME J 13:847–859. doi:10.1038/s41396-018-0319-2. 30504898 PMC6461938

[62] Piqué N, Miñana-Galbis D, Merino S, Tomás JM. 2015. Virulence factors of *Erwinia amylovora*: a review. Int J Mol Sci 16:12836–12854. doi:10.3390/ijms160612836. 26057748 PMC4490474

[63] Rering CC, Beck JJ, Hall GW, McCartney MM, Vannette RL. 2018. Nectar-inhabiting microorganisms influence nectar volatile composition and attractiveness to a generalist pollinator. New Phytol 220:750–759. doi:10.1111/nph.14809. 28960308

[64] Martiny AC, Treseder K, Pusch G. 2013. Phylogenetic conservatism of functional traits in microorganisms. ISME J 7:830–838. doi:10.1038/ismej.2012.160. 23235290 PMC3603392

[65] Zavala-Gonzalez EA, Rodríguez-Cazorla E, Escudero N, Aranda-Martinez A, Martínez-Laborda A, Ramírez-Lepe M, Vera A, Lopez-Llorca LV. 2017. *Arabidopsis thaliana* root colonization by the nematophagous fungus *Pochonia chlamydosporia* is modulated by jasmonate signaling and leads to accelerated flowering and improved yield. New Phytol 213:351–364. doi:10.1111/nph.14106. 27456071

[66] Vaingankar JD, Rodrigues BF. 2012. Screening for efficient AM (arbuscular mycorrhizal) fungal bioinoculants for two commercially important ornamental flowering plant species of Asteraceae. Biol Agric Hortic 28:167–176. doi:10.1080/01448765.2012.727541.

[67] Liu S, Guo H, Xu J, Song Z, Song S, Tang J, Chen X. 2018. Arbuscular mycorrhizal fungi differ in affecting the flowering of a host plant under two soil phosphorus conditions. J Plant Ecol 11:623–631. doi:10.1093/jpe/rtx038.

[68] Fan Y, Luan Y, An L, Yu K. 2008. Arbuscular mycorrhizae formed by *Penicillium pinophilum* improve the growth, nutrient uptake and photosynthesis of strawberry with two inoculum-types. Biotechnol Lett 30:1489–1494. doi:10.1007/s10529-008-9691-8. 18483699

[69] Cifuentes-Arenas JC, de Goes A, de Miranda MP, Beattie GAC, Lopes SA. 2018. Citrus flush shoot ontogeny modulates biotic potential of *Diaphorina citri*. PLoS One 13:e0190563. doi:10.1371/journal.pone.0190563. 29304052 PMC5755881

[70] Pons E, Peris JE, Peña L. 2012. Field performance of transgenic citrus trees: assessment of the long-term expression of uidA and nptII transgenes and its impact on relevant agronomic and phenotypic characteristics. BMC Biotechnol 12:41. doi:10.1186/1472-6750-12-41. 22794278 PMC3462728

[71] Gilbert J. 2012. The Earth Microbiome Project: a new paradigm in geospatial and temporal studies of microbial ecology. SciVee.

[72] Lundberg DS, Yourstone S, Mieczkowski P, Jones CD, Dangl JL. 2013. Practical innovations for high-throughput amplicon sequencing. Nat Methods 10:999–1002. doi:10.1038/nmeth.2634. 23995388

[73] Blaustein RA, Lorca GL, Meyer JL, Gonzalez CF, Teplitski M. 2017. Defining the core citrus leaf- and root-associated microbiota: factors associated with community structure and implications for managing Huanglongbing (Citrus Greening) disease. Appl Environ Microbiol 83. doi:10.1128/AEM.00210-17. PMC5440699 28341678

[74] McMurdie PJ, Holmes S. 2013. phyloseq: an R package for reproducible interactive analysis and graphics of microbiome census data. PLoS One 8:e61217. doi:10.1371/journal.pone.0061217. 23630581 PMC3632530

[75] Oksanen J, Kindt R, Legendre P, O’Hara B, Stevens MHH, Oksanen MJ, Suggests M. 2007. The vegan package. Community Ecology Package 10:631–637.

[76] Hervé M. 2015. RVAideMemoire: diverse basic statistical and graphical functions. R package version 0.9–45-2. R Foundation for Statistical Computing, Vienna, Austria.

[77] Lahti L, Shetty S, Blake T, Salojarvi J. 2017. Microbiome r package. R Foundation for Statistical Computing, Vienna, Austria.

[78] Letunic I, Bork P. 2016. Interactive tree of life (iTOL) v3: an online tool for the display and annotation of phylogenetic and other trees. Nucleic Acids Res 44:W242–W245. doi:10.1093/nar/gkw290. 27095192 PMC4987883

[79] Love M, Anders S, Huber W. 2014. Differential analysis of count data–the DESeq2 package. boiRxiv https://www.biorxiv.org/content/10.1101/002832v3.

[80] Ginestet C. 2011. ggplot2: elegant graphics for data analysis. J R Stat Soc 174:245–246. doi:10.1111/j.1467-985X.2010.00676_9.x.

[81] Kurtz ZD, Müller CL, Miraldi ER, Littman DR, Blaser MJ, Bonneau RA. 2015. Sparse and compositionally robust inference of microbial ecological networks. PLoS Comput Biol 11:e1004226. doi:10.1371/journal.pcbi.1004226. 25950956 PMC4423992

