# Supplementary figures and images for: Microbial Turnover and Dispersal Events Occur in Synchrony with Plant Phenology in the Perennial Evergreen Tree Crop Citrus sinensis
Source: mBio. 2022 Jun 1;13(3):e00343-22. doi: 10.1128/mbio.00343-22 (PMC9239260; doi:10.1128/mbio.00343-22)

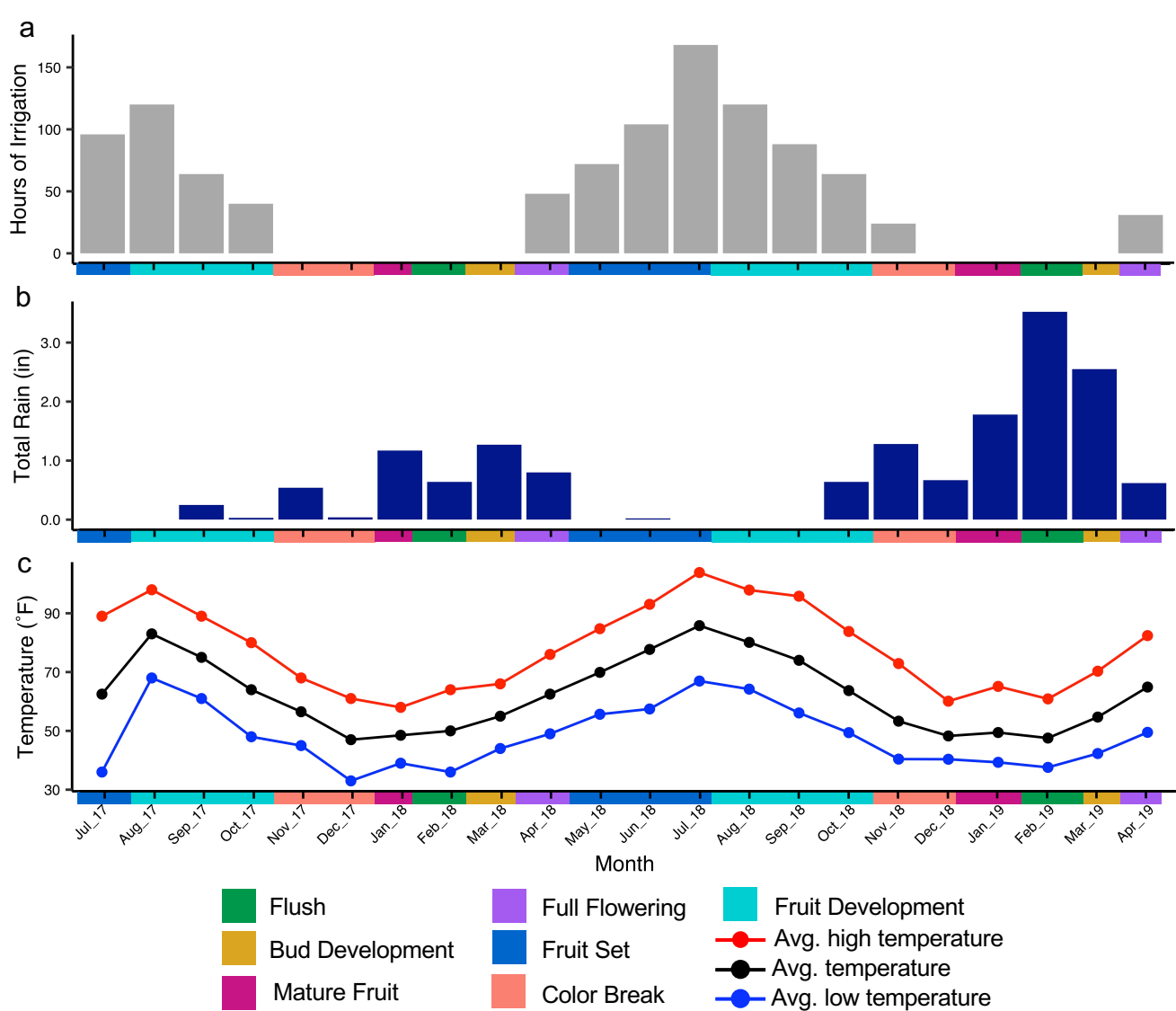

Supplement: FIG S1 [file mbio.00343-22-s0001.pdf]

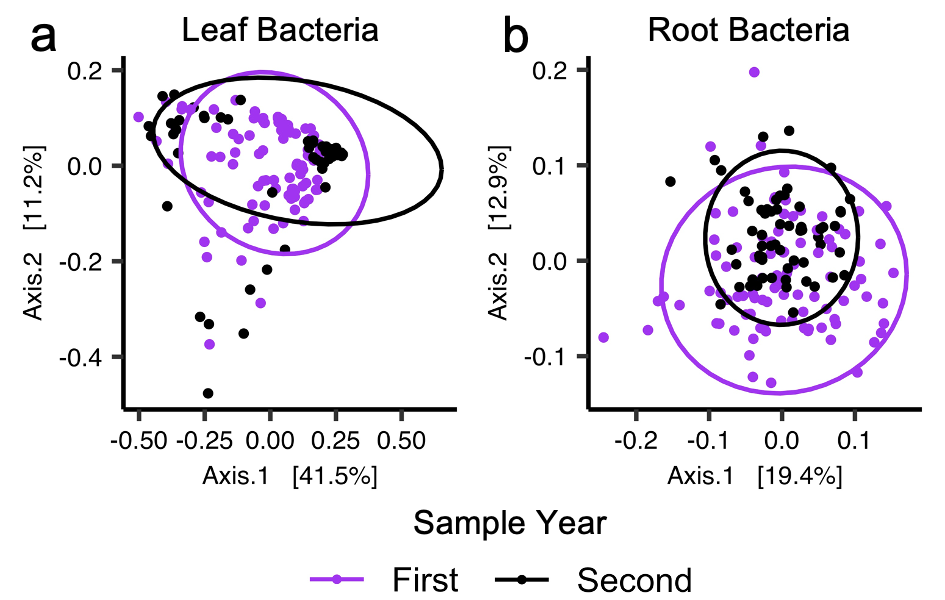

Supplement: FIG S2 [file mbio.00343-22-s0002.tif]

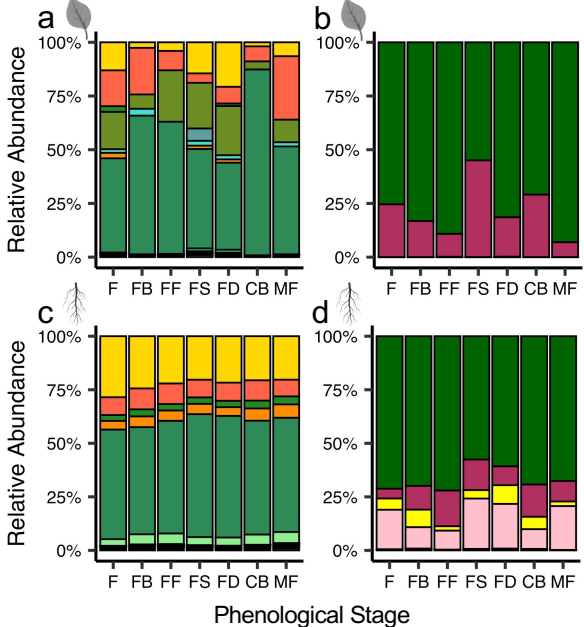

Supplement: FIG S3 [file mbio.00343-22-s0003.pdf]

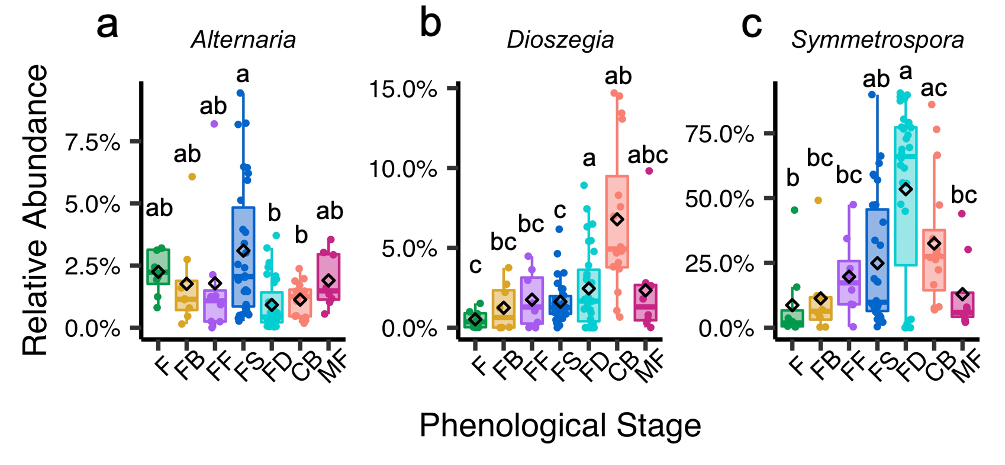

Supplement: FIG S5 [file mbio.00343-22-s0005.tif]
