# Supplementary material for: Microbial Turnover and Dispersal Events Occur in Synchrony with Plant Phenology in the Perennial Evergreen Tree Crop Citrus sinensis
Source: mBio. 2022 Jun 1;13(3):e00343-22. doi: 10.1128/mbio.00343-22 (PMC9239260; doi:10.1128/mbio.00343-22)
Supplement: TABLE S2 [file mbio.00343-22-s0007.docx]

| **Phenological Stage Pairwise-PERMANOVA Results** | | | | |
| --- | --- | --- | --- | --- |
| **Comparison** | **Leaf Bacteriome** | **Root Bacteriome** | **Leaf Mycobiome** | **Root Mycobiome** |
| F X CB | **0.002** | 0.356 | **0.009** | 0.338 |
| F X FB | **0.002** | 0.188 | 0.882 | 0.498 |
| F X FD | **0.002** | 0.090 | **0.008** | 0.066 |
| F X FF | **0.002** | 0.082 | **0.020** | 0.261 |
| F X FS | **0.002** | 0.056 | **0.034** | 0.070 |
| F X MF | **0.019** | 0.202 | **0.028** | 0.517 |
| FB X CB | **0.009** | 0.089 | **0.009** | 0.165 |
| FB X FD | **0.003** | **0.021** | **0.007** | 0.066 |
| FB X FF | **0.007** | 0.625 | **0.039** | 0.965 |
| FB X FS | **0.002** | **0.033** | 0.150 | 0.056 |
| FB X MF | **0.003** | 0.283 | **0.034** | 0.088 |
| FF X CB | **0.002** | **0.029** | **0.008** | 0.084 |
| FF X FD | **0.002** | **0.029** | **0.007** | **0.050** |
| FF X FS | **0.002** | **0.050** | **0.008** | **0.042** |
| FF X MF | **0.009** | 0.277 | **0.007** | **0.047** |
| FS X CB | **0.002** | **0.033** | **0.007** | **0.042** |
| FS X FD | **0.002** | **0.021** | **0.007** | 0.498 |
| FS X MF | **0.002** | 0.121 | **0.015** | 0.202 |
| FD X CB | **0.002** | **0.029** | **0.008** | **0.042** |
| FD X MF | **0.004** | 0.061 | **0.007** | 0.202 |
| MF X CB | **0.035** | 0.283 | **0.034** | 0.165 |
