# Supplementary material for: Microbial Turnover and Dispersal Events Occur in Synchrony with Plant Phenology in the Perennial Evergreen Tree Crop Citrus sinensis
Source: mBio. 2022 Jun 1;13(3):e00343-22. doi: 10.1128/mbio.00343-22 (PMC9239260; doi:10.1128/mbio.00343-22)
Supplement: FIG S4 [file mbio.00343-22-s0004.pdf]

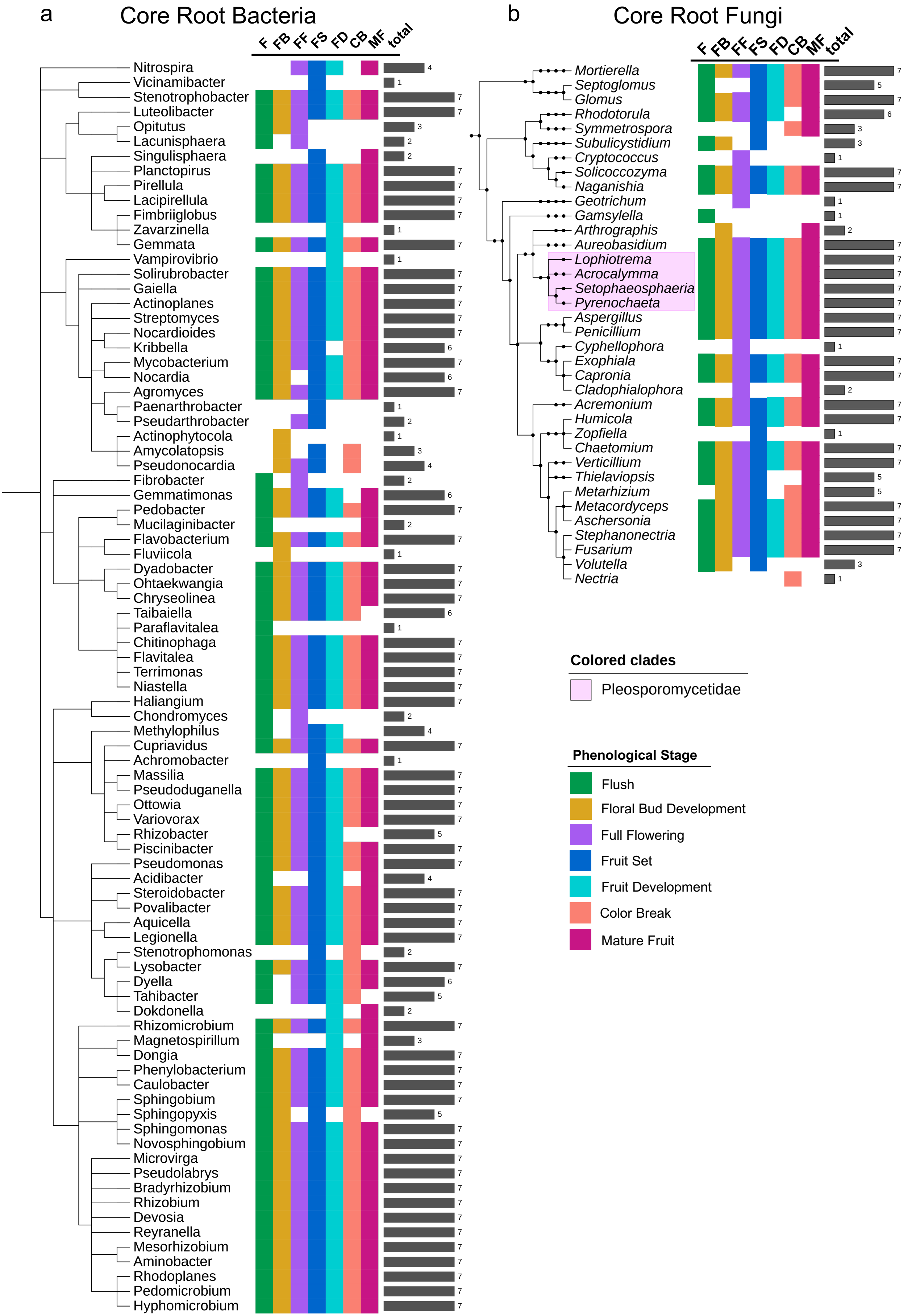

a
Core Root Bacteria
F FB FF FS FD CB MF total
Nitrospira
Vicinamibacter
Stenotrophobacter
Luteolibacter
Opitutus
Lacunisphaera
Singulisphaera
Planctopirus
Pirellula
Lacipirellula
Fimbriiglobus
Zavarzinella
Gemmata
Vampirovibrio
Solirubrobacter
Gaiella
Actinoplanes
Streptomyces
Nocardioides
Kribbella
Mycobacterium
Nocardia
Agromyces
Paenarthrobacter
Pseudarthrobacter
Actinophytocola
Amycolatopsis
Pseudonocardia
Fibrobacter
Gemmatimonas
Pedobacter
Mucilaginibacter
Flavobacterium
Fluviicola
Dyadobacter
Ohtaekwangia
Chryseolinea
Taibaiella
Paraflavitalea
Chitinophaga
Flavitalea
Terrimonas
Niastella
Haliangium
Chondromyces
Methylophilus
Cupriavidus
Achromobacter
Massilia
Pseudoduganella
Ottowia
Variovorax
Rhizobacter
Piscinibacter
Pseudomonas
Acidibacter
Steroidobacter
Povalibacter
Aquicella
Legionella
Stenotrophomonas
Lysobacter
Dyella
Tahibacter
Dokdonella
Rhizomicrobium
Magnetospirillum
Dongia
Phenylobacterium
Caulobacter
Sphingobium
Sphingopyxis
Sphingomonas
Novosphingobium
Microvirga
Pseudolabrys
Bradyrhizobium
Rhizobium
Devosia
Reyranella
Mesorhizobium
Aminobacter
Rhodoplanes
Pedomicrobium
Hyphomicrobium
b
Core Root Fungi
F FB FF FS FD CB MF total
Mortierella
Septoglomus
Glomus
Rhodotorula
Symmetrospora
Subulicystidium
Cryptococcus
Solicoccozyma
Naganishia
Geotrichum
Gamsylella
Arthrographis
Aureobasidium
Lophiotrema
Acrocalymma
Setophaeosphaeria
Pyrenochaeta
Aspergillus
Penicillium
Cyphellophora
Exophiala
Capronia
Cladophialophora
Acremonium
Humicola
Zopfiella
Chaetomium
Verticillium
Thielaviopsis
Metarhizium
Metacordyceps
Aschersonia
Stephanonectria
Fusarium
Volutella
Nectria
Colored clades
Pleosporomycetidae
Phenological Stage
Flush
Floral Bud Development
Full Flowering
Fruit Set
Fruit Development
Color Break
Mature Fruit
